# EsxN drives ISG15-mediated dsDNA release to activate cGAS-STING signaling and promote mycobacterial survival

**DOI:** 10.1128/spectrum.02488-25

**Published:** 2026-02-25

**Authors:** Qiao Zhang, Abulimiti Abudukadier, Haiqi Chen, Luyan Xiong, Peibo Li, Zhen Gong, Jianping Xie

**Affiliations:** 1 State Key Laboratory Breeding Base of Eco-Environment and Bio-Resource of the Three Gorges Area, Key Laboratory of Eco-environments in Three Gorges Reservoir Region, Ministry of Education, School of Life Sciences, Institute of Modern Biopharmaceuticals, Southwest University 26463, Chongqing, China; 2 Chongqing Public Health Medical Center 567508, Chongqing, China; 3 Department of Clinical Laboratory, The Second Affiliated Hospital of Anhui Medical University 533251, Hefei, Anhui, China; Tainan Hospital Ministry of Health and Welfare, Tainan, Taiwan

**Keywords:** EsxN, *Mycobacterium*, type I interferon, ISG15, cGAS-STING

## Abstract

Tuberculosis, caused by *Mycobacterium tuberculosis*, remains a major global health threat largely due to the pathogen’s remarkable ability to subvert host immune defenses. A key mechanism of this subversion is the type VII secretion systems, particularly the Esx-5 subsystem. The Esx-5 secretion system secretes substrates that are hypothesized to function as effector proteins, orchestrating host immune modulation and influencing the outcome of *M. tuberculosis* infections. While EsxN is a confirmed secreted effector of ESX-5, its precise biological function remains unknown. Our research aims to fill this gap by investigating how EsxN manipulates host innate immunity, particularly through the cGAS-STING axis. EsxN was overexpressed in *Mycobacterium smegmatis* (Ms_EsxN) using an acetamide-inducible vector. Wild-type and ISG15-deficient THP-1 macrophages were infected with Ms_EsxN or control strains (MOI = 10). Bacterial intracellular survival was quantified by CFU assay. Host cell viability was assessed via the CCK-8 kit. Cytosolic double-stranded DNA (dsDNA) was visualized using PicoGreen staining and confocal microscopy. Transcriptomes of infected cells were analyzed by RNA-seq. Type I interferon (IFN-I)/cytokine responses and pathway activation were validated by RT-qPCR and immunoblotting (TBK1/phospho-TBK1 [p-TBK1], IRF3, ISG15, IL-1β). ISGylation was detected by ubiquitin-like modifier blotting. Statistical significance was determined by Student’s *t*-test (*P* < 0.05). We demonstrate that the Esx-5 substrate EsxN (Rv1793) is a key *M. tuberculosis* virulence effector that drives immune evasion by hijacking the cGAS-STING-ISG15 axis. EsxN overexpression exhibited enhanced intracellular survival in human macrophages without affecting host viability. Crucially, EsxN triggers increased dsDNA leakage into the cytoplasm, amplifies IFN-I responses, and suppresses pro-inflammatory cytokines, enhancing bacterial intracellular survival. ISG15 deficiency abolished EsxN-driven effects, revealing that ISG15-dependent ISGylation is essential for this immune evasion. Our study uncovers a novel immune evasion strategy wherein *M. tuberculosis* exploits host DNA-sensing machinery to amplify immunosuppressive IFN-I responses via EsxN-ISG15 crosstalk, which suggests targeting ISG15 as a potential host-directed therapy against tuberculosis.

**IMPORTANCE:**

Tuberculosis (TB) remains one of the world’s deadliest infectious diseases, killing over a million people annually. A major challenge in treating TB is understanding how the *Mycobacterium tuberculosis* evades our immune defenses to survive inside human cells. This study reveals that an *M. tuberculosis* protein, EsxN, acts as a “molecular trickster” by hijacking a key immune pathway-cGAS-STING and weakens the body’s ability to control TB infection, allowing the bacteria to survive better. Crucially, we identified ISG15 as an essential partner in this evasion strategy. These findings expose a hidden weapon used by *M. tuberculosis* and highlight ISG15 or DNA leakage as promising targets for new host-directed therapies.

## INTRODUCTION

Tuberculosis (TB), a historically significant human disease, continues to represent a leading cause of global mortality. The etiological agent of TB*, Mycobacterium tuberculosis* (Mtb), primarily targets macrophages, the cornerstone of innate immunity against pathogens. *Mycobacterium marinum* (Mm), a close relative of *M. tuberculosis*, serves as an exceptional model organism for studying TB pathogenesis. Notably, Mm exhibits a high degree of genetic and functional homology with Mtb, sharing approximately 85% genomic identity and utilizing conserved virulence mechanisms to infect and manipulate host macrophages. These bacteria employ sophisticated mechanisms to establish infection, evade host defenses, and subvert macrophage functions.

*M. tuberculosis* is a highly successful pathogen. Its outer membrane, rich in mycolic acids, exhibits exceptionally low permeability (1). To transport virulence proteins across this impermeable membrane, *M. tuberculosis* has evolved specialized type VII secretion systems (T7SS). Structural comparisons with known bacterial secretion systems reveal that the type VII system is fundamentally distinct, indicating a unique secretion mechanism (2). The genomic architecture of *M. tuberculosis* underscores the critical importance of the T7SS for its pathogenesis. The presence of multiple, conserved Esx loci (Esx-1 to Esx-5) within its genome is not redundant; rather, it signifies a sophisticated evolutionary adaptation. Among these, the Esx-5 system, found exclusively in pathogenic mycobacteria, has emerged as a central player in modulating the host-pathogen interface. Substrates secreted by this system regulate bacterial survival, host immune responses, and nutrient metabolism (3).

The Esx secretion system enables bacterial adaptation to the harsh environment of the phagolysosome, thereby facilitating bacterial escape and nutrient acquisition (4). The virulence mediated by Esx-1 is attributed to its ability to damage the membrane of the macrophage phagosomal compartment where the bacterium resides (5). Most T7SS substrates belong to three protein families: Esx, PE, and PPE proteins. Notably, in pathogenic mycobacteria, the number of *pe* and *ppe* genes is extensively expanded, accounting for nearly 10% of the coding capacity (6). *Mycobacterium smegmatis* (Ms), a non-pathogenic, fast-growing mycobacterium commonly used as a model for studying mycobacterial physiology, possesses the Esx-1, Esx-3, and Esx-4 subsystems. However, Esx-2 and Esx-5 systems are found only in slow-growing mycobacteria. Successful reconstitution of the Esx-5 system in *M. smegmatis* demonstrated that heterologous assembly requires five membrane components: EccB5, EccC5, EccD5, EccE5, and MycP5 (3). Although both Esx-2 and Esx-5 are missing in *M. smegmatis*, the Esx-5 system is unique and conserved in pathogenic mycobacteria and is indispensable for their full virulence, primarily through the secretion of multiple effector proteins that modulate host immunity. *M. tuberculosis* activates ESX-5 secretion in response to inorganic phosphate (P_i_) limitation (7). RegX3 is a response regulator activated during P_i_ limitation, directly activating the transcription of the esx-5 gene subset, enhancing the secretion of EsxN and PPE41 substrates, and assisting *Mycobacterium tuberculosis* in replication during infection (8–11). Early proteomics studies have confirmed that EsxN is a substrate secreted by the Esx-5 pathway, which strongly suggests that it plays a specific role in host-pathogen interactions. Unlike some antigens that are prone to mutation, EsxN shows a high degree of sequence conservation in clinical isolates (12), which suggests, from an evolutionary perspective, that its function may be of great significance for the adaptability or survival of bacteria. At present, most of the research on EsxN focuses on its potential as a candidate antigen for subunit vaccines. However, as an effector protein, how it directly regulates the host cell immune signaling pathway during infection remains a significant knowledge gap.

Following *M. tuberculosis* infection of macrophages, the ESAT-6/CFP-10 complex secreted by Esx-1 system disrupts the phagosomal membrane (4). This disruption leads to the leakage of double-stranded DNA (dsDNA) into the cytosol. Cytosolic dsDNA is recognized by cyclic GMP-AMP synthase (cGAS), which catalyzes the production of the second messenger cyclic GMP-AMP (cGAMP). cGAMP subsequently activates the STING-TBK1-IRF3 signaling axis, driving the transcription of type I interferons (IFNs), such as IFN-β. Among the T7SS of *M. tuberculosis*, the Esx-5 subsystem has attracted significant attention, second only to the extensively studied Esx-1. To date, the confirmed substrates of Esx-5 include several highly homologous Esx proteins and numerous PE/PPE family proteins. Substrates of this family have been demonstrated to assist *M. tuberculosis* in escaping from phagosomes and to play critical roles in mycobacterial-host interactions. The Esx-5-secreted substrate, like PE_PGRS47, can prolong cytosolic DNA accumulation by inhibiting autophagolysosomal degradation (13, 14). Heterologous expression of the *M. marinum* ESX-1 region in BCG (*Mycobacterium bovis* BCG) induced the cGAS/STING/TBK1/IRF-3/type I interferon axis in infected mice and enhanced AIM2 and NLRP3 inflammasome activity. This immune activation resulted in a higher proportion of multifunctional CD4 Th1 cells specific for ESX-1 antigens and CD8 T cell effectors targeting shared mycobacterial antigens of BCG (15).

Research indicates that the cGAS-STING axis is activated not only by non-self DNA (e.g., from DNA viruses, retroviruses, or intracellular pathogens like bacteria and protozoa) but also by host-derived DNA (including mitochondrial and nuclear DNA) that enters the cytosol (16). Cytosolic DNA potently activates type I interferon responses, although the precise mechanisms remain incompletely understood (17). Under physiological conditions, DNA is primarily confined to the nucleus and mitochondria, and it can be rapidly degraded by nucleases in the cytoplasm and endolysosomes. During infection, intracellular DNA accumulation increases (18, 19). TBK1 in patients with spinal tuberculosis has higher specificity, and IFN-β in serum has higher sensitivity when used to distinguish between patients with active and stable lesions (20). Paradoxically, while type I IFNs (e.g., IFN-α, IFN-β) are critical antiviral effectors, they suppress antibacterial signaling pathways (21, 22) and promote infection by numerous intracellular bacteria, including *M. tuberculosis* (23, 24) and *Listeria monocytogenes* (25–27). This suggests that bacterial pathogens exploit this antiviral pathway for their benefit. Significantly, studies have demonstrated that cGAS directly binds the *M. tuberculosis* genomic DNA during macrophage infection, providing *in vivo* evidence of interaction between an *M. tuberculosis* ligand and a host pattern recognition receptor (17). Collectively, T7SS play crucial roles in mycobacterial growth, virulence, and pathogenesis. Understanding the molecular mechanisms by which T7SS substrates engage host immunity could therefore pave the way for novel therapeutic strategies against tuberculosis.

## MATERIALS AND METHODS

### Bacterial and cell culture

*M. smegmatis* mc² 155, Ms_pAL, and Ms_EsxN were cultured in Middlebrook 7H9 liquid medium containing 0.2% glycerol as a carbon source with 20 μg/mL hygromycin and 0.05% Tween-80 as a dispersing agent, or on Middlebrook 7H10 solid medium supplemented with 2% glycerol. *Escherichia coli* DH5α was cultivated in Luria-Bertani medium. Wild-type THP-1 and ISG15-deficient THP-1 cells were cultured in RPMI-1640 medium supplemented with 10% fetal bovine serum (FBS, Gibco, 10091148), 2 mM L-glutamine, 100 μg/mL streptomycin, and 100 U/mL penicillin (Invitrogen) at 37°C under 5% CO_2_.

### Generation of mouse bone marrow-derived macrophages

Bone marrow cells were flushed from tibiae and femurs with PBS, filtered through a 70 μm cell strainer, resuspended in DMEM supplemented with 10% FBS and 30% L929 cell-conditioned medium (as a source of macrophage colony-stimulating factor) and incubated for 6 days at 37°C, 5% CO_2_. Bone marrow-derived macrophage (BMDM) cultures were then washed with PBS and detached with 1% trypsin, and 5 × 10^5^ cells were seeded into each well at (using 24-well plates).

### Construction of recombinant bacteria

The coding region of EsxN was amplified from the genomic DNA of *M. tuberculosis* H37Rv (The *M. tuberculosis* H37Rv genome was obtained from Beijing Chest Hospital, Capital Medical University) and subsequently cloned into the pALACE plasmid to generate pALACE-EsxN. Both the pALACE-EsxN and the empty pALACE control plasmid were electroporated into wild-type *M. smegmatis* mc² 155. Electroporated cells were plated on Middlebrook 7H10 agar with 50 μg/mL hygromycin for 3–5 days to produce Ms_EsxN and Ms_pAL strains, respectively.

### Immunofluorescence and confocal microscopy

Cells were cultured on 15 mm glass-bottom confocal dishes. At specified infection time points, culture supernatant was discarded, and cells were fixed with 4% paraformaldehyde for 20 min at room temperature. After washing with PBS, cells were incubated with PicoGreen dye (Yeasen) at the recommended concentration for 10 minutes according to the manufacturer’s instructions, followed by PBS washes to remove excess dye. For the LysoTracker staining assay, cells were cultured in Lyso-Tracker Red (Beyotime) for 30 min without fixing and PBS washed for three times. For the MitoTracker staining assay, cells were cultured in MitoTracker Green FM (Thermo Fisher) for 30 min. Furthermore, cells were washed with PBS and fixed with 4% paraformaldehyde for 10 minutes at room temperature. Cells were visualized using the confocal microscope. Confocal images were acquired using an Olympus laser scanning confocal microscope (model specifics: FLUOVIEW FV3000) and analyzed using ZEN software (Zeiss). For each experimental condition, we analyzed a robust sample from a minimum of three distinct fields of view to ensure statistical power. All comparative imaging was performed with identical instrumental settings.

### Immunoblot analysis

Cells were infected with different bacterial strains at a multiplicity of infection (MOI) of 10:1. At 6 h, 24 h, and 48 h post-infection, cells were washed three times and lysed using RIPA buffer to collect total protein. Proteins were separated by SDS-PAGE (15% or 18% gels) and subsequently transferred to nitrocellulose membranes at 15 V for 1 h. Membranes were blocked with 5% bovine serum albumin or 5% non-fat dry milk in TBST (Tris-buffered saline with 0.1% Tween-20) for 2 h at room temperature with gentle shaking. Membranes were then probed with primary antibodies against TBK1, p-TBK1, IRF3, ISG15, STAT1, p-STAT1, IL-1β, LAMP1, ACTIN (β-actin), and p-P65 (phospho-NF-κB p65). After five washes with TBST, membranes were incubated with appropriate horseradish peroxidase-conjugated secondary antibodies (goat anti-mouse or goat anti-rabbit) for 2 h at room temperature. Protein bands were visualized using an ECL chemiluminescence kit.

### Small interfering RNA gene silencing

THP-1 cells were plated at 1 × 10^6^ cells per milliliter in 12-well plates for 48 h with 100 ng/mL PMA. On the day of transfection, the media were replaced with 900 μL 1640 without penicillin/streptomycin. Then, THP-1 cells were incubated with 100 μL siRNA and CALNPYM RNAi *in vitro* transfection reagent (DN-001-10, Beijing, China). Twenty-four hours after transfection, cells were infected with different strains.

### Bacterial infection and CFU assay

THP-1 cells or BMDMs were seeded in 12-well plates at a density of 1 × 10⁶ cells/well, and THP-1 cells were differentiated with 0.1 µg/mL phorbol 12-myristate 13-acetate (PMA) for 48 h. BMDMs and PMA-differentiated THP-1 macrophages were infected with Ms_EsxN or Ms_pALACE at an MOI of 10:1. After 4 h, cells were washed three times with sterile PBS and re-incubated with fresh medium containing 10% FBS, supplemented with gentamicin at a final concentration of 100 µg/mL to kill extracellular bacteria. For CFU enumeration, macrophages were lysed in PBS containing 0.025% SDS. Lysates collected at various time points were serially diluted in PBS and plated onto 7H10 agar plates. After 3–4 days of incubation at 37°C, bacterial colonies were counted.

### Growth curve analysis

Growth curves for both *M. smegmatis* strains were assessed spectrophotometrically using a Varian Cary 50 UV-Vis spectrophotometer. Briefly, overnight bacterial cultures were diluted into 7H9 liquid medium to an optical density (OD_600_) of 0.8. A 1% inoculum was then transferred to fresh 7H9 liquid medium containing 50% acetamide to induce EsxN expression. Cultures were incubated at 37°C with shaking at 110 rpm. OD_600_ was measured every 4 h until the bacteria reached the stationary phase.

### Bacterial biofilm and single colony culture

#### Biofilm culture

In a 12-well plate, prepare 2 mL per well of 7H9 liquid culture medium supplemented with 20 μL acetamide, glucose to a final concentration of 1%, and 20 μL Tween-80 solution (concentration if known). Add the bacterial suspension at the desired proportion to the corresponding wells (include inoculation density/OD). Perform replicates for each sample (*n* = 3). Incubate in a 37°C constant-temperature incubator for 2–3 days. Observe biofilm formation.

#### Single colony morphology

Dilute the adjusted bacterial suspension (OD_600_ ≈ 0.8) to 10^−5^. Spread 100 μL of the diluted bacterial solution evenly onto the surface of 7H10 solid culture medium. Incubate plates at 37°C in a constant-temperature incubator for 3–5 days. Observe colony morphology.

### RNA sequencing

Total RNA was extracted from WT THP-1 cells infected with Ms_EsxN or Ms_pAL strains for 48h using the TRIzol reagent method. RNA quality was assessed, and qualified RNA was used to prepare paired-end (PE) libraries according to the manufacturer’s protocol of the VAHTS Universal V6 RNA-seq Library Prep Kit for Illumina (Vazyme). Briefly, mRNA was purified from 1 μg of total RNA using oligo(dT) magnetic beads, followed by fragmentation in ABclonal First Strand Synthesis Reaction Buffer. Using the fragmented mRNA as a template, first-strand cDNA was synthesized with random primers and reverse transcriptase. Second-strand cDNA was then synthesized using DNA Polymerase I, RNase H, buffer, and dNTPs. The resulting double-stranded cDNA fragments were ligated with adapters and amplified by PCR. PCR products were purified, and library quality was assessed using an Agilent Bioanalyzer 4150. Finally, sequencing was performed on an Illumina NovaSeq 6000/MGISEQ-T7 platform (Shanghai Applied Protein Technology Co., Ltd., Shanghai, China).

### RT-qPCR

At 24 h and 48 h post-infection with the respective bacterial strains, total cellular RNA was extracted from infected BMDMs and THP-1 cells using an RNA extraction kit (Promega) following the manufacturer’s instructions. cDNA synthesis was performed using a PrimeScript RT reagent kit (Takara, Shiga, Japan). Quantitative real-time RT-PCR reactions were carried out on a CFX96 RT-PCR detection system (Bio-Rad) using SYBR Green Master Mix. Relative mRNA levels were calculated by normalization to β-actin (ACTB) and GAPDH expression using the 2^(-ΔΔCt)^ method.

### Cell viability and cell death assay

For cell viability assessment, cells were seeded in 96-well plates and evaluated using a CCK-8 cell counting kit (Solarbio, CCK-8) according to the manufacturer’s instructions. Absorbance was measured at 450 nm.

### Statistical analysis and biological replicates

All experiments were performed with a minimum of three independent biological replicates. Data from replicate experiments were analyzed using Student’s *t*-test to ensure result reliability. For data visualization, software such as GraphPad Prism, ImageJ, and R was used to present data distributions and trends via box plots, bar graphs, or line graphs for initial comparisons. Data are presented as the mean ± standard error of the mean from at least three independent experiments. Statistical analyses were performed using GraphPad Prism 8.0. Results from RT-qPCR and CFU assays were analyzed using Student’s *t*-test. Differences were considered statistically significant when the *P*-value was less than 0.05 (*P* < 0.05), highly significant when *P* < 0.01, and extremely significant when *P* < 0.001.

## RESULTS

### EsxN potentially functions as a virulence factor influencing mycobacterial immune evasion capability

**Fig 1:**
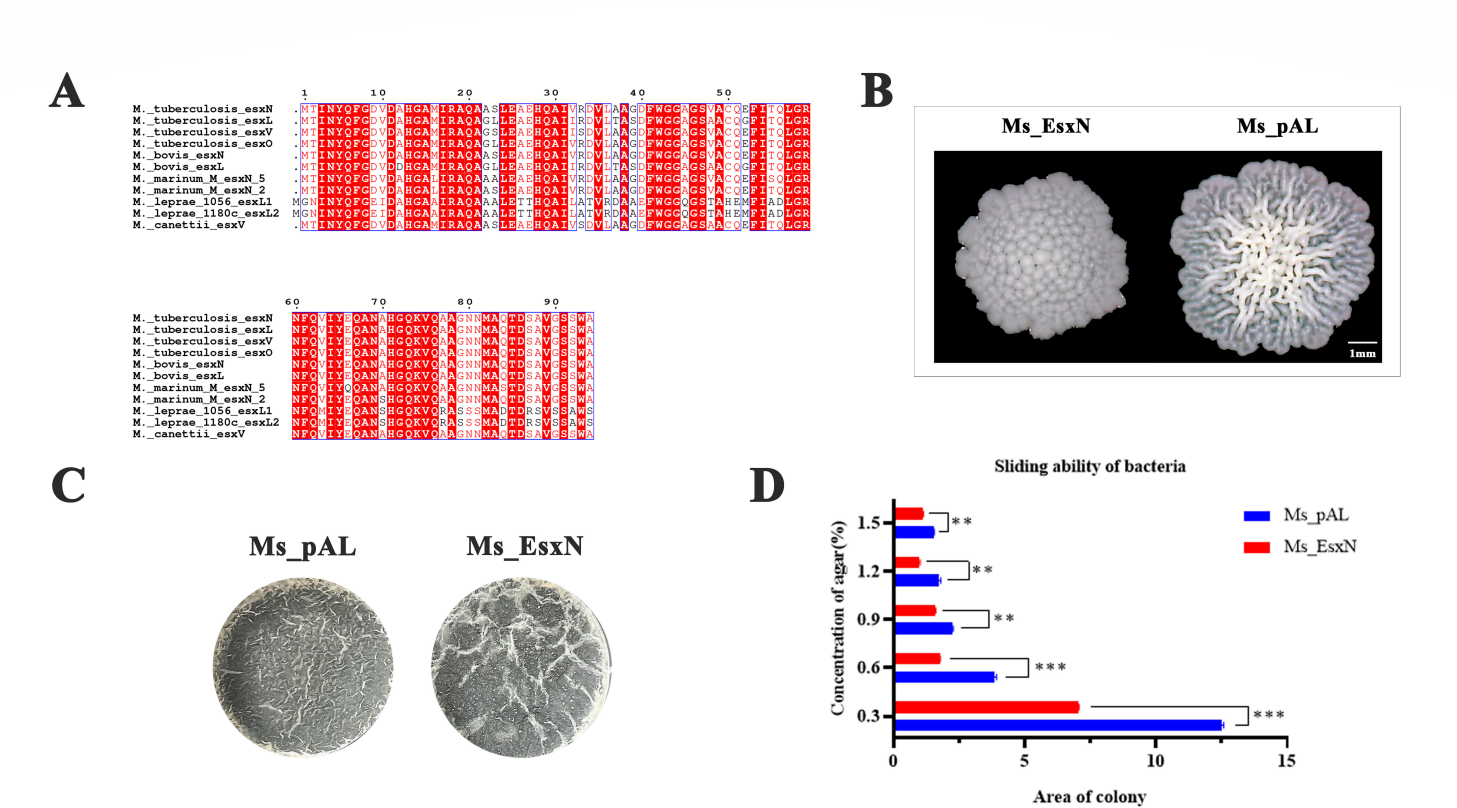
EsxN is involved in influencing the mycobacterial cell envelope. (**A**) Amino acid sequence alignment of EsxN and its homologous genes’ amino acid sequences in *Mycobacterium*. (**B**) Ms_pAL and Ms_EsxN were inoculated onto Colombian blood plates at extremely low concentrations, and the morphology of individual *M. smegmatis* colonies was photographed after 5 days. (**C**) Inoculated Ms_pAL and Ms_EsxN into 7H9 liquid medium containing 20% glucose and free of Tween-80, and incubated in the dark for 3–4 days to observe the biofilm formation ability of *M. smegmatis*. (**D**) Ms_pAL and Ms_EsxN were inoculated into 7H9 plates containing 0.3%, 0.6%, 0.9%, 1.2%, and 1.5% agarose, and their sliding ability on the plates was observed as *M. smegmatis* grew. Error bars represent standard deviation; (*P ≤ 0.05, **P ≤ 0.01, ***P ≤ 0.001; *n* = 3).

As one of the Esx-5 substrates, EsxN (Rv1793) has been identified as a potential subunit vaccine candidate. Amino acid sequence alignment revealed strong conservation of EsxN across mycobacterial species (Fig. 1A), suggesting functional conservation and highlighting its research significance. To further investigate its biological role, we overexpressed *M. tuberculosis* EsxN in *M. smegmatis* using 50% acetamide (ACE) induction (Fig. S1). On 7H9 solid agar plates, EsxN altered mycobacterial colony morphology, decreasing surface wrinkling and rendering the edges smoother (Fig. 1B). In biofilm assays cultured in 7H9 medium supplemented with 20% glucose, EsxN altered biofilm architecture, and overexpression of EsxN promoted enhanced wrinkling in mycobacterial biofilms (Fig. 1C). Additionally, Ms_EsxN also exhibited markedly reduced sliding motility versus the Ms_pAL control on 7H9 agarose-gradient plates (Fig. 1D). These findings suggest that EsxN remodels the cell envelope of *M. smegmatis*. Given the pivotal role of lipids in *M. tuberculosis* virulence, we propose that EsxN may function as a key effector modulating mycobacterial pathogenicity.

### EsxN facilitates immune evasion of recombinant mycobacteria from THP-1 cells

**Fig 2:**
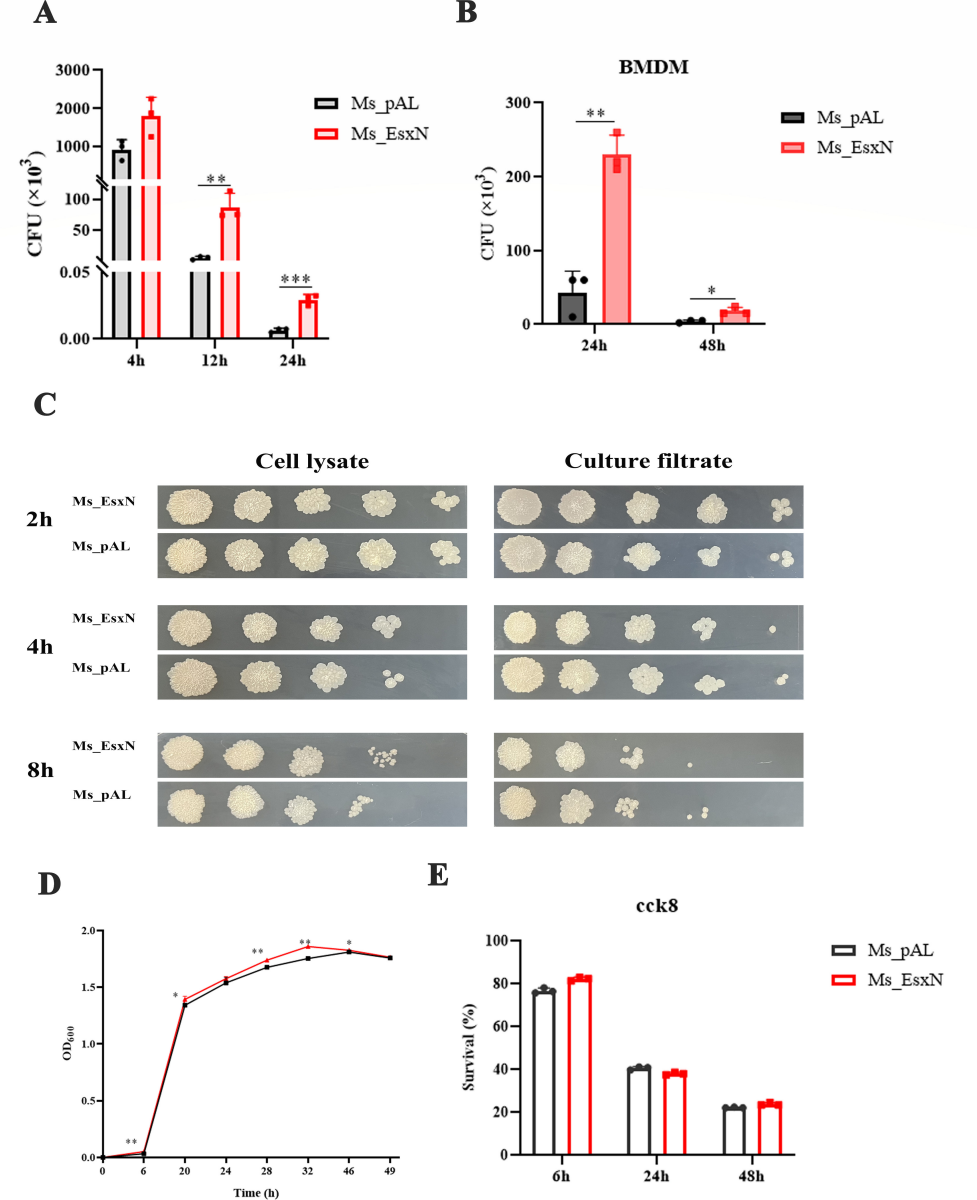
EsxN facilitates immune evasion of recombinant mycobacteria from THP-1 cells. (**A, B**) The intracellular survival rates of Ms_pAL and Ms_EsxN in THP-1 and BMDMs at 4 h, 12 h, 24 h, and 48 h time points. (**C**) Mycobacterial burden in the cell culture supernatant and cell lysate of THP-1 cells infected for 2 h, 4 h, and 8 h was cultured and grown on 7H9 solid culture dishes. (**D**) The growth ability of Ms_pAL and Ms_EsxN in 7H9 liquid medium. (**E**) When THP-1 cells were infected with Ms_pAL and Ms_EsxN for 6 h, 24 h, and 48 h, CCK-8 reagent was added to the 96-well plate. The absorbance value at 450 nm was detected by a microplate reader to evaluate the viability of the cells. All error bars represent standard deviation. (*P ≤ 0.05, **P ≤ 0.01, ***P ≤ 0.001; *n* = 3).

To further validate whether EsxN genuinely participates in influencing host cell immune responses and mycobacterial immune evasion capability, we firstly simulated the acidic environment of phagosomes and lysosomes in mycobacterial 7H9 medium and found that Ms_EsxN is more tolerant in acidic environments with pH = 3 than Ms_pAL (Fig. S2), which indicates Ms-EsxN may have a stronger survival ability in phagolysosomes than Ms_pAL. Then we established infection models using wild-type THP-1 cells infected with either Ms_pAL or Ms_EsxN. PMA-differentiated THP-1 macrophages were infected at an MOI of 10 for 4 h, 12 h, and 24 h, and the bacterial load within host cells was quantified. We found that EsxN significantly enhanced mycobacterial survival within host cells (Fig. 2A). We infected mouse BMDMs with the two strains, respectively, and found that the CFU level in BMDMs was significantly higher than that in the control group after EsxN overexpression (Fig. 2B). To further elucidate the mechanism by which EsxN enhances intracellular survival of *M. smegmatis*, we quantified the mycobacterial load in both cell lysates and cell culture supernatants at 2 h, 4 h, and 8 h post-infection. We found that EsxN overexpression did not affect macrophage phagocytic capacity or mycobacterial invasive ability, and the bacterial loads inside and outside the cells were comparable (Fig. 2C). Consequently, the ability of macrophages to phagocytose, digest, and lyse mycobacteria early in infection is not impacted by EsxN overexpression. Crucially, growth curve analysis confirmed that EsxN overexpression did not affect intrinsic bacterial growth kinetics (Fig. 2D), indicating that the observed increase in bacterial load was not due to intrinsic growth differences between Ms_pAL and Ms_EsxN, but rather resulted from EsxN’s involvement in mycobacterial-host cell interactions. Subsequently, host cell viability was assessed post-infection using CCK-8 assays. No significant differences in THP-1 cell survival rates were detected at 6 h, 24 h, or 48 h (Fig. 2E). These data collectively demonstrate that EsxN-mediated immune evasion results from direct enhancement of bacterial persistence mechanisms, independent of modulation of host cell death pathways.

### EsxN induces a stronger IFN-I response and antagonizes host inflammatory immune responses

**Fig 3:**
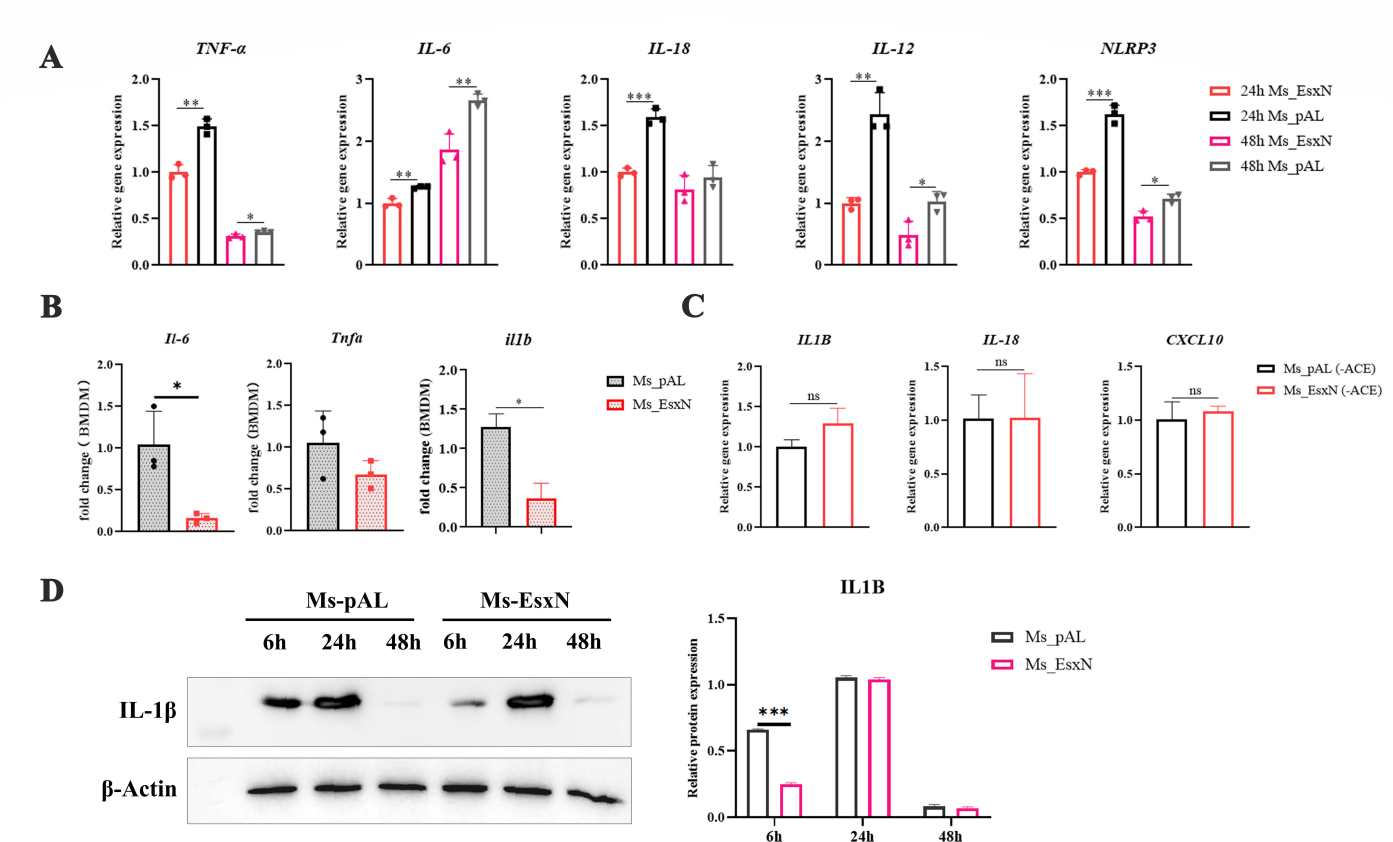
EsxN weakens the pro-inflammatory immune response of THP-1 cells. (**A**) The pro-inflammatory immune level of the host and the transcriptional activation of the inflammasome NLRP3 at the time points of 24 h and 48 h after THP-1 cell infection were detected by RT-qPCR. (**B**) The RT-qPCR method was used to detect the pro-inflammatory cytokines of the host at 24 h time points in BMDMs infected with *M. smegmatis*. (**C**) The RT-qPCR method was used to detect the pro-inflammatory immune levels of the host at 24 h and 48 h time points in THP-1 cells infected with *M. smegmatis* without EsxN expression induced by 50% ACE. (**D**) Western blotting was used to detect the expressional levels of IL-1β in THP-1 cells infected with Ms_pAL and Ms_EsxN for 6 h, 24 h, and 48 h. The gray values of the protein bands were analyzed using ImageJ. All error bars represent standard deviation; (*P ≤ 0.05, **P ≤ 0.01, ***P ≤ 0.001; ****P ≤ 0.0001; *n* = 3; ns, not significant).

The survival capability of mycobacteria within host cells is often closely linked to the inflammatory immune response elicited by infection. To investigate the molecular mechanism by which EsxN overexpression aids mycobacterial immune evasion, we first examined the inflammatory response in THP-1 cells infected with Ms_pAL or Ms_EsxN. Compared to Ms_pAL-infected THP-1 cells, infection with Ms_EsxN significantly downregulated the transcription levels of both classic inflammatory cytokines of innate immunity and the core inflammasome component NLRP3 at 24 h and 48 h time points (Fig. 3A). We also found that the expression of pro-inflammatory cytokines was inhibited by overexpression of EsxN in primary mouse BMDMs (Fig. 3B). When EsxN expression is not induced by an inducer, there is no significant difference in the transcriptional levels of the related genes (Fig. 3C). Immunoblotting results demonstrated that IL-1β expression levels were indeed lower in the Ms_EsxN infection group compared to the Ms_pAL group (Fig. 3D). This indicates that EsxN dampens the host’s pro-inflammatory immune response, providing a more stable environment for mycobacterial survival within host cells and reducing immune-mediated killing.

**Fig 4:**
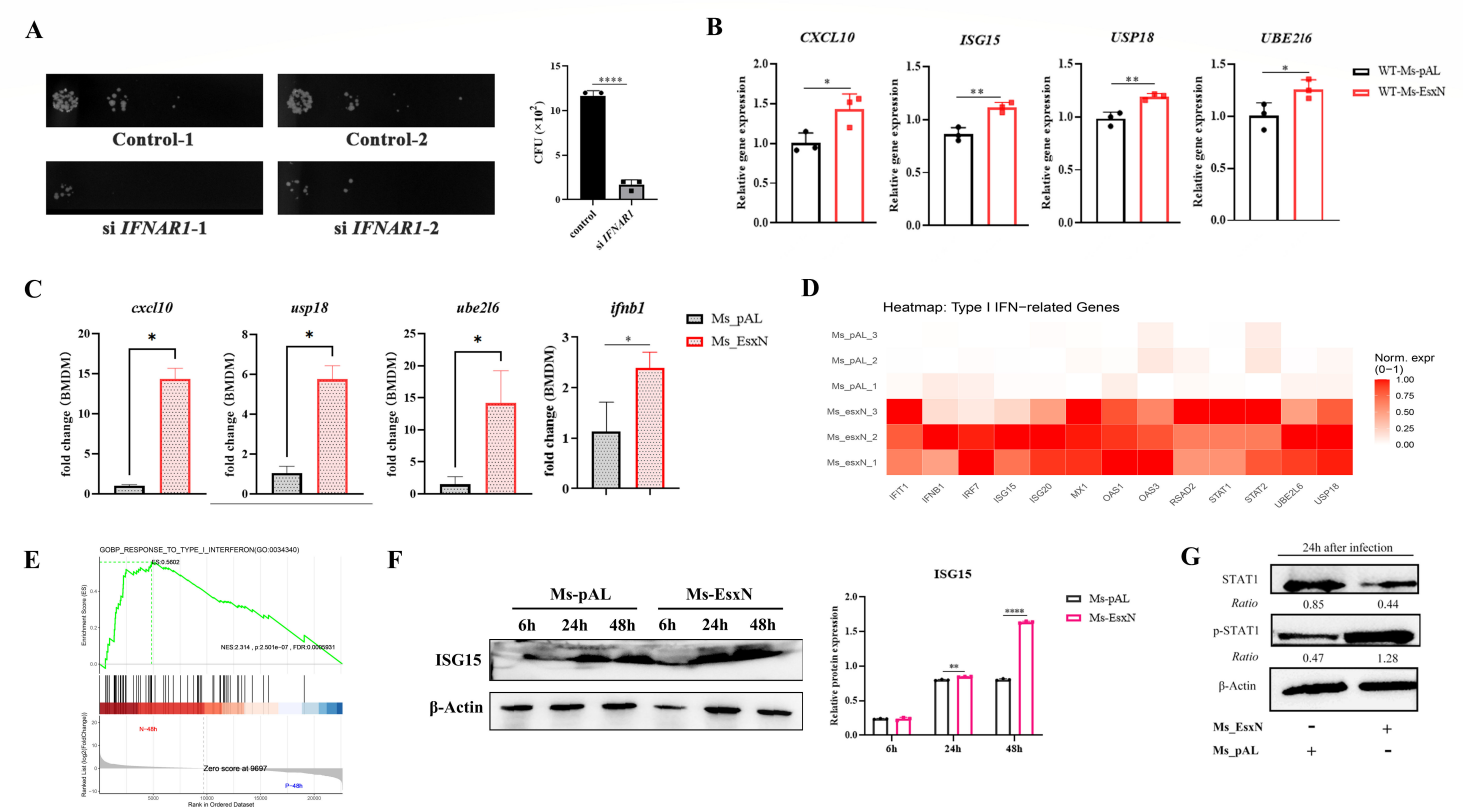
EsxN elevated the type I interferon response of THP-1 macrophages. (**A**) CFU levels were detected in THP-1 knockdown cells 24 h after IFNAR1 silencing. (**B and C**) The type I interferon response level of the host in THP-1 cells and BMDMs infected for 24 h was detected by RT-qPCR. (**D and E**) The expression heatmap of type I interferon-related genes and GSEA were plotted using R Studio (version 4.4.1). We used the dplyr software package for GO function enrichment analysis. When *P* < 0.05, it is considered that the GO or KEGG function is significantly enriched. DEGs with |log2FC| > 1 and *P*_adj_ < 0.05 were considered to be significantly different expressed genes. (**F**) Western blotting was used to detect the expressional levels of ISG15 in THP-1 cells infected with Ms_pAL and Ms_EsxN for 6 h, 24 h, and 48 h. (**G**) Western blotting was used to detect the expressional levels of STAT1 and p-STAT1 in THP-1 cells infected with Ms_pAL and Ms_EsxN for 24 h. The gray values of the protein bands were analyzed using ImageJ. All error bars represent standard deviation; (*P ≤ 0.05, **P ≤ 0.01, ***P ≤ 0.001; ****P ≤ 0.0001; *n* = 3).

In individuals infected with *M. tuberculosis*, macrophages present antigens to T cells, which subsequently secrete IFN-γ to activate macrophages and mediate innate immune responses. Analysis of two tuberculosis patients’ data sets revealed that *M. tuberculosis* infection substantially upregulates the IFN-I response (28) (Fig. S3A and B). Research indicates that this phenotype promotes the expression levels of IL-10 and IL-1Ra, thereby suppressing IL-1α/β levels and impairing host control of *M. tuberculosis* (29). Therefore, we knocked down *IFNAR1* in THP-1 cells, the upstream receptor of the type I interferon pathway, to block the type I interferon pathway. After 24 h of infection with *M. smegmatis*, we found that the survival rate of *M. smegmatis* was significantly reduced in the knocked-down cells (Fig. 4A), proving that type I interferon is indeed detrimental to the host’s clearance of mycobacteria. Further validation using RT-qPCR to detect the transcription of related genes in infected THP-1 cells confirmed, consistent with the transcriptomics data, that EsxN induced higher levels of transcription for genes associated with the type I interferon response (Fig. 4B). Consistently, the levels of type I interferon-responsive genes and *Ifnb* (interferon-β) were significantly increased in primary mouse BMDMs (Fig. 4C). GAPDH, as a second housekeeping gene for RT-qPCR, was detected by RT-qPCR (Fig. S3C). Consequently, we performed transcriptomic analysis on THP-1 cells infected with Ms_pAL or Ms_EsxN for 48 h. Based on the gene transcription expression profiles, we found that the transcription levels of type I interferon-related genes were significantly upregulated (Fig. 4D). Gene set enrichment analysis (GSEA) further enriched biological processes in THP-1 cells to the type I interferon response pathway (Fig. 4E). Elevated protein expression levels of ISG15 and p-STAT1 in the Ms_EsxN infection group also corroborated the activation of type I interferon signaling (Fig. 4F and G). Altogether, these data indicate that EsxN upregulates the host type I interferon response to antagonize the intracellular pro-inflammatory immune response. To further confirm whether this inhibitory effect of EsxN on pro-inflammatory cytokines is an indirect effect induced by IFN-I or a directly driven event by EsxN, we knocked down *IFNAR1* (interferon alpha receptor 1). We found that the expression of *IL1B* (Interleukin-1 beta) was higher in the Ms_EsxN group than in the Ms_pAL group after knockdown of this gene, indicating that EsxN is not a direct factor driving the reduction of host pro-inflammatory cytokines, but type I interferon (Fig. S3D).

### EsxN mediates the release of more cytosolic dsDNA to activate cGAS-STING and induce ISGs transcription

**Fig 5:**
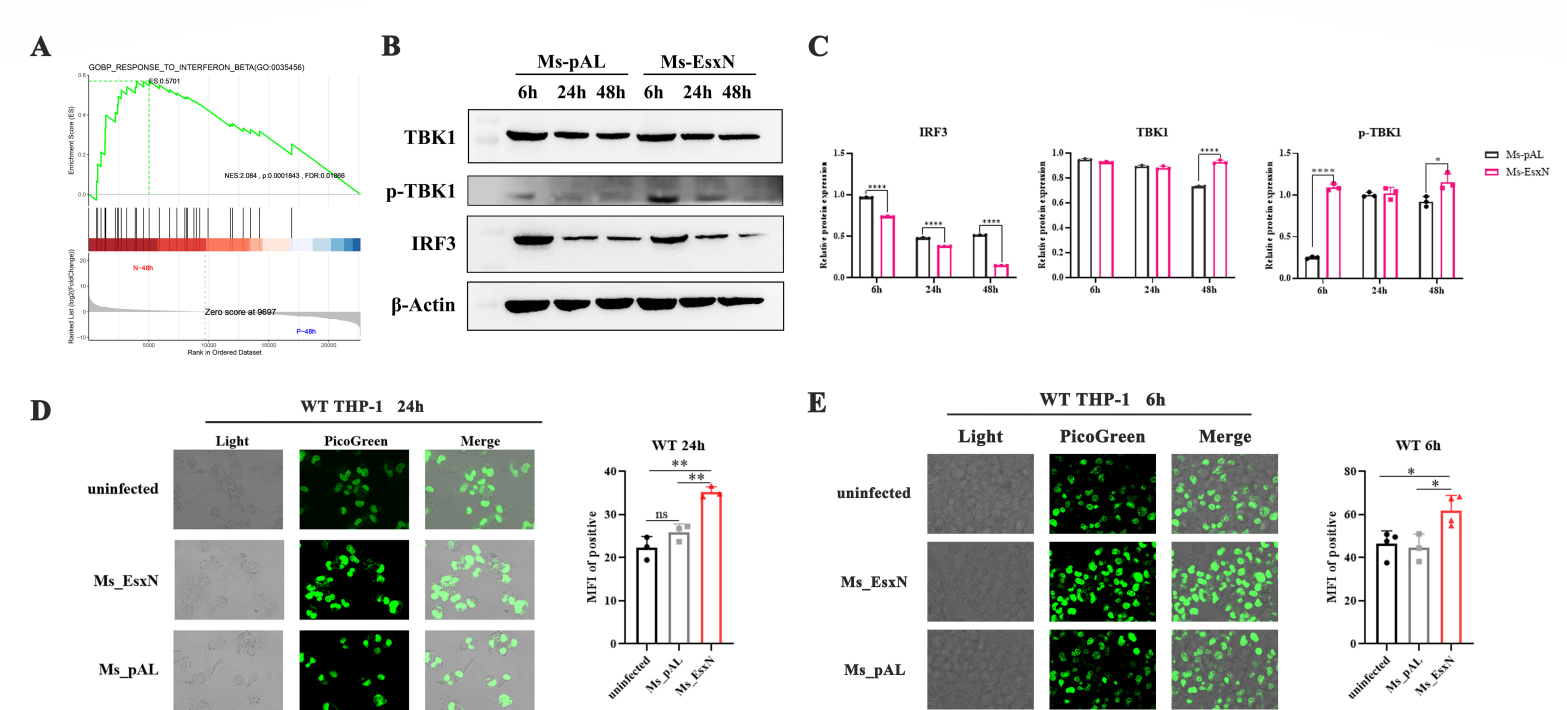
EsxN mediates the release of more cytosolic dsDNA to activate the cGAS-STING pathway. (**A**) We used the dplyr software package for “RESPONSE OF INTERFERON BETA” GO function enrichment analysis. When *P* < 0.05, it is considered that the GO function is significantly enriched. (**B**) Western blotting was used to detect the expressional levels of TBK1, p-TBK1, and IRF3 in THP-1 cells infected with Ms_pAL and Ms_EsxN for 6 h, 24 h, and 48 h. (**C**) Statistical analysis of the protein amounts of TBK1, p-TBK1, and IRF3 in THP-1 macrophages. The relative amounts of TBK1, p-TBK1, and IRF3 were normalized to β-actin. All error bars represent standard deviation; (*P ≤ 0.05, **P ≤ 0.01, ***P ≤ 0.001; ****P ≤ 0.0001; *n* = 3). (**D, E**) Immunostaining of the content of intracellular double-stranded DNA 6 h and 24 h after infection, which was captured by confocal fluorescence microscopy (100×). PicoGreen is a green fluorescent dye that binds to dsDNA. Statistical analysis of the mean fluorescence intensity (MFI) in ImageJ. All error bars represent standard deviation; (*P ≤ 0.05, **P ≤ 0.01, ***P ≤ 0.001; ****P ≤ 0.0001; *n* = 3).

The discovery of the cGAS-STING pathway represents a major milestone in innate immunity, sensing both exogenous pathogen-derived dsDNA and endogenous cytosolic dsDNA. Transcriptomics data revealed that differential GSEA also enriched the IFN-β response pathway (Fig. 5A). Upregulation of Ifnb transcription is a key downstream indicator of cGAS-STING signaling pathway activation. We therefore hypothesized that EsxN might mediate the observed increase in downstream type I interferon signaling and enhanced intracellular survival by influencing the activation of the cGAS-STING pathway. To test this, we assessed the protein expression levels of TBK1 and p-TBK1 in THP-1 cells infected with Ms_pAL or Ms_EsxN at 6 h, 24 h, and 48 h time points using immunoblotting. The results showed that EsxN overexpression promoted TBK1 phosphorylation (Fig. 5B and C). Furthermore, the basal expression level of IRF3 protein was lower in the Ms_EsxN group compared to the Ms_pAL group, indicating activation of the cGAS-STING pathway by EsxN (Fig. 5B and C). To further validate whether EsxN mediates cGAS-STING activation, we utilized PicoGreen, a highly sensitive dsDNA fluorescent dye, and confirmed elevated cytosolic dsDNA in EsxN-infected THP-1 cells at 24 h by confocal microscopy (Fig. 5D). These data mechanistically link EsxN to cGAS-STING pathway potentiation through dsDNA accumulation and downstream signaling activation.

Given the early onset of TBK1 activation during infection, we further quantified cytosolic dsDNA levels at the 6 h post-infection. Similarly, stronger green fluorescence was observed in Ms_EsxN-infected THP-1 cells (Fig. 5E). Taken together, these results indicate that EsxN can induce cells to produce more dsDNA as early as the initial stages of infection, thereby activating the cGAS-STING signaling pathway to induce the transcription of downstream ISGs.

**Fig 6:**
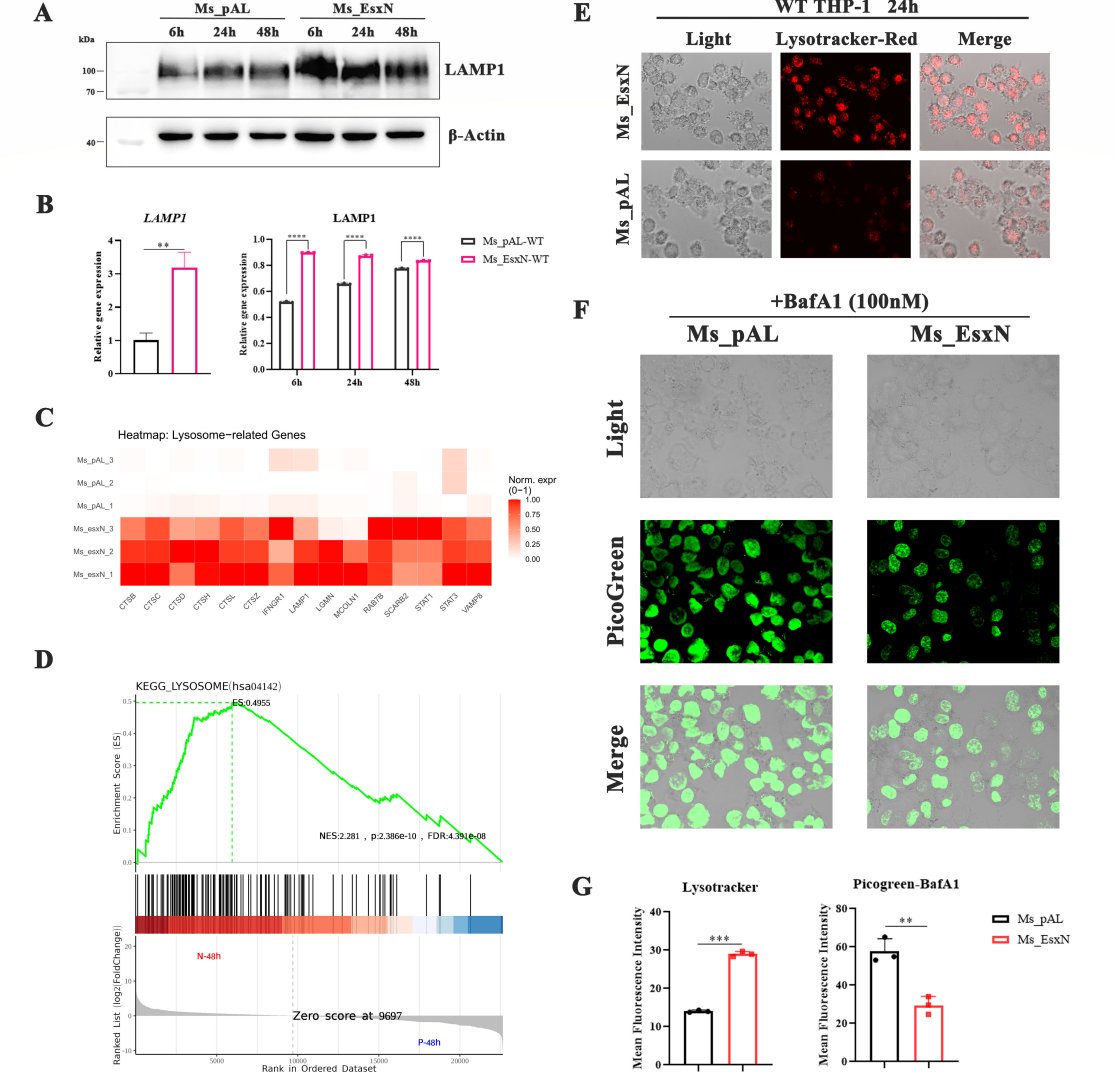
EsxN promotes the leakage of cytoplasmic dsDNA by influencing lysosomal biogenesis. (**A**) Western blotting was used to detect the expression levels of LAMP1 in THP-1 cells infected with Ms_pAL and Ms_EsxN for 6 h, 24 h, and 48 h. (**B**) The transcriptional level of *LAMP1* in THP-1 cells infected 24 h prior was detected by RT-qPCR. Statistical analysis of the protein amounts of LAMP1 in THP-1 macrophages. The relative amounts of LAMP1 were normalized to β-actin. (**C and D**) The expression heatmap of lysosome-related genes and GSEA was plotted using R Studio (version 4.4.1). We used the dplyr software package for GO function enrichment analysis. When *P* < 0.05, it is considered that the GO or KEGG functions were significantly enriched. (**E–G**) Immunostaining of the content of intracellular lysosomes and double-stranded DNA 24 h after infection, which was captured by confocal fluorescence microscopy (100×). (**F**) The cells were infected with MS in the presence of bafilomycin A1 (BafA1, 100 nM) for 3 h. Statistical analysis of the mean fluorescence intensity (MFI) of LysoTracker and PicoGreen staining in cells infected with MS and treated with BafA1 in ImageJ. All error bars represent standard deviation; (*P ≤ 0.05, **P ≤ 0.01, ***P ≤ 0.001; ****P ≤ 0.0001; *n* = 3).

However, the precise source and mechanism for the accumulation of cytosolic dsDNA remained unclear. Given that the integrity of lysosomes is crucial for maintaining cytoplasmic homeostasis and that lysosomal membrane permeabilization can lead to the leakage of contents, including self-DNA, into the cytosol, we hypothesized that EsxN might contribute to dsDNA release by compromising lysosomal function. Preliminary detection of the lysosomal membrane protein LAMP1 showed that EsxN overexpression induced higher transcription and expression of LAMP1 protein (Fig. 6A and B). Transcriptome analysis also revealed significant upregulation of lysosome-related genes, and GSEA enriched the lysosomal pathway (Fig. 6C and D), implying that EsxN-induced lysosomal stress (supported by LAMP1 upregulation and lysosomal gene enrichment) facilitates dsDNA leakage into the cytosol. We assessed lysosomal status using LysoTracker staining. Our investigations showed that the EsxN-overexpressing group exhibited a robustly increased LysoTracker fluorescence intensity compared to the pALACE control group (Fig. 6E). To directly test whether this lysosomal alteration contributed to the cytosolic dsDNA, we inhibited lysosomal acidification using bafilomycin A1 (BafA1). Subsequent quantification of cytosolic dsDNA revealed a markedly reduced level in the EsxN group under BafA1 treatment (Fig. 6F and G). These results indicate that the process of EsxN enhancing the number of cytoplasmic double-stranded DNA involves lysosomes, which strongly implicates the lysosome as one of the primary sources contributing to the increased cytosolic dsDNA pool observed during EsxN-mediated infection.

### EsxN activates the cGAS-STING pathway involving ISG15

**Fig 7:**
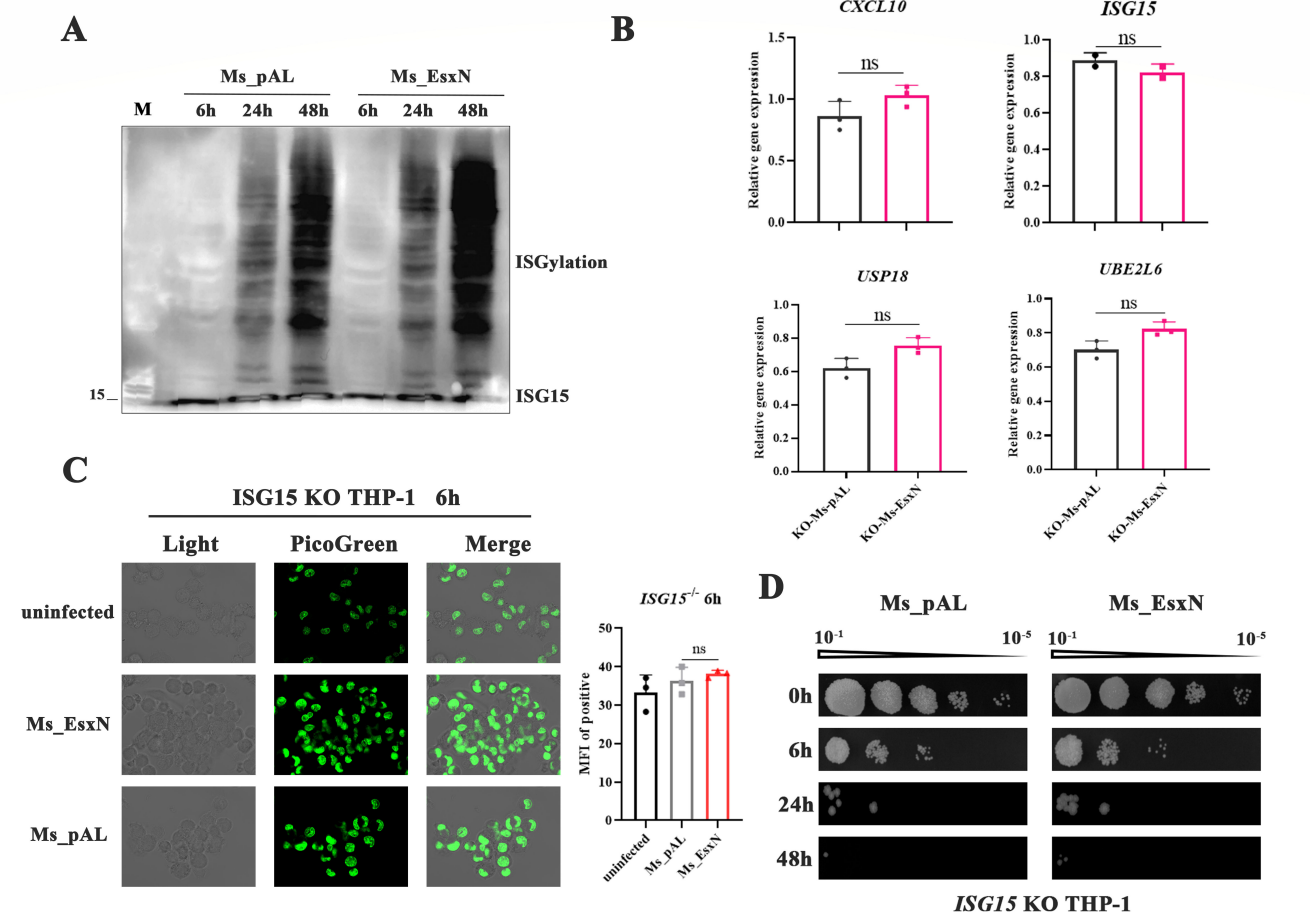
The release of cytoplasmic dsDNA and the activation of cGAS-STING require the participation of ISG15. (**A**) Western blotting was used to detect the expression levels of ISG15 and ISGylation in THP-1 cells infected with Ms_pAL and Ms_EsxN for 6 h, 24 h, and 48 h. (**B**) RT-qPCR was used to detect the transcriptional levels of host inflammatory factors in ISG15 KO THP-1 cells infected with Ms_pAL and Ms_EsxN for 24 h. All error bars represent standard deviation; (*P ≤ 0.05, **P ≤ 0.01, ***P ≤ 0.001; *n* = 3; ns, not significant). (**C**) Immunostaining of the content of intracellular double-stranded DNA in ISG15 KO THP-1 at 6 h after infection, which was captured by confocal fluorescence microscopy (100×). PicoGreen is a green fluorescent dye that binds to dsDNA. (**D**) The intracellular survival ability of Ms_pAL and Ms_EsxN in ISG15 KO THP-1 cells at 0 h, 6 h, 24 h, and 48 h time points.

ISG15, a ubiquitin-like modifier, regulates STING and cGAS activity through ISGylation (30), thereby modulating DNA sensing and antiviral immunity (31). Moreover, consistent with our transcriptomic data, ISG15 was upregulated in Ms_EsxN-infected THP-1 cells (Fig. 4F), promoting the hypothesis that ISG15 might play a critical role in EsxN-mediated host immune responses. Immunoblotting analysis demonstrated significant enhancement of ISG15 and global ISGylated conjugates in EsxN-infected cells, with maximal induction at 48 h post-infection (Fig. 7A). Subsequently, immunoblotting confirmed the successful gene knockout of ISG15 (Fig. S3F). Furthermore, we infected ISG15-deficient THP-1 cells with either Ms_pAL or Ms_EsxN. We observed that the difference in host type I interferon response induced by EsxN overexpression disappeared at 24 h post-infection (Fig. 7B). Confocal microscopy detection of cytosolic dsDNA levels revealed no significant difference and a trend towards consistent levels in ISG15-deficient THP-1 cells infected for 6 h (Fig. 7C). This indicates that ISG15 is required for EsxN-induced high levels of dsDNA and subsequent cGAS-STING activation. We further assessed the intracellular survival of both strains in ISG15-deficient cells and found that the survival advantage conferred by EsxN was abolished (Fig. 7D), demonstrating that ISG15 deficiency suppresses the enhanced *M. smegmatis* survival mediated by EsxN overexpression.

## DISCUSSION

Macrophages are the natural host cells for *M. tuberculosis*. During infection, *M. tuberculosis* secretes substrate virulence proteins into the cell membrane or extracellular space via T7SS, which then interact with various host proteins, forming a complex pathogen-host interaction network. Therefore, elucidating the molecular interactions by which *M. tuberculosis* virulence proteins engage host immune responses is crucial for understanding TB pathogenesis. After being phagocytosed, *M. tuberculosis* damages its phagosomal compartment to enter the host cell cytosol. In the absence of Esx-1, bacteria are confined to the phagosomal compartment (32, 33). Our study demonstrates that EsxN induces macrophages to release more dsDNA into the cytosol. Moreover, studies have indicated that lysosomal biogenesis plays a significant role in the increase of cytosolic dsDNA (34).

Given the complexity and dynamic homeostasis of the intracellular environment, the origins of cytosolic dsDNA are multifaceted and closely tied to immune and metabolic pathways, warranting a preliminary discussion of the various possibilities. The sources of cytosolic dsDNA are diverse, including dsDNA released from the nucleus and damaged mitochondria (35, 36), or dsDNA leaked due to altered lysosomal membrane stability caused by substrate overload (34), or dsDNA released under lysosomal stress conditions. When bacteria infect macrophages, they are engulfed by phagosomes generated by the macrophages. These phagosomes subsequently fuse with lysosomes and mature into phagolysosomes. The ability of SMAD-specific E3 ubiquitin protein ligase 1 (SMURF1) to recruit microtubule-associated protein 1 light chain 3 beta 2 and LAMP1 is crucial for phagophore formation, which is necessary for bacterial xenophagy in *M. tuberculosis* (4). ESAT-6-like protein EsxP can block phagosome maturation, facilitating bacterial escape into the cytosol to avoid the hostile acidic environment (37). These findings collectively underscore the critical impact of T7SS substrates on subverting host phagocytic defenses. We found that EsxN potentially modulates lysosomal biogenesis, as its overexpression led to augmented lysosomal numbers and/or acidity (Fig. 6E). We posit that this lysosomal alteration may directly contribute to the release of cytosolic dsDNA, a compelling hypothesis that will be a central direction of our subsequent investigations.

**Fig 8:**
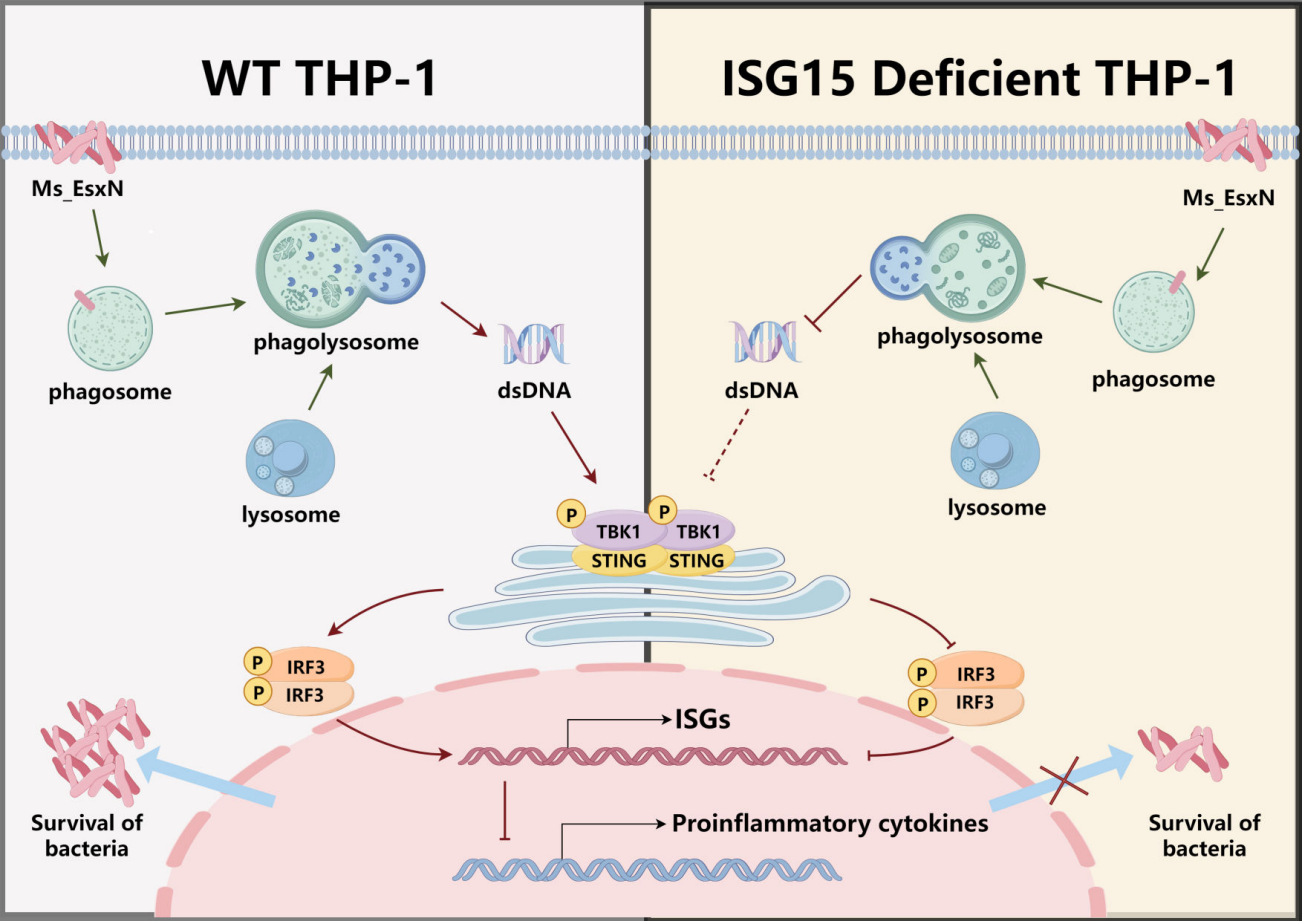
The molecular mechanism of EsxN immune escape from macrophage killing and the role of ISG15 in this process. In wild-type THP-1 cells, when infected by EsxN-overexpressed *M. smegmatis*, the bacteria are phagocytosed by phagosomes and further mature to form phagolysosomes. EsxN induces the cells to release more dsDNA to further activate the cGAS-STING signaling pathway. After IRF3 translocation to the nucleus, it activates the transcription of downstream type I interferon-stimulated genes, which in turn antagonizes the transcriptional activation of pro-inflammatory cytokines, ultimately preventing mycobacteria from being killed, providing them with a more stable survival environment, and promoting the survival of mycobacteria. However, with ISG15 deficiency, EsxN cannot induce cells to release more dsDNA to activate the cGAS-STING signaling pathway, and the transcriptional level of type I interferon-stimulated genes cannot be activated as a result, finally leading to the disappearance of the mycobacterial survival advantage brought by EsxN.

While in our study, we did not delve into the potential leakage of nuclear or mitochondrial DNA, infection may influence host cell apoptosis, leading to the formation and lysis of apoptotic bodies that release dsDNA (35). When apoptosis is inhibited, STING activation can also promote RIPK3-dependent necrotic apoptosis by participating in type I interferon and TNF signaling pathways (38). Lymphocytes and monocytes seem to die preferentially after STING activation (39–42). However, preliminary assessment of apoptosis-related markers indicated that EsxN had minimal impact on apoptosis, and Annexin V-FITC/PI double staining also suggested it may not be a mechanism of host cell death (Fig. S4). Third, mitochondrial DNA (mtDNA) represents a major genetic component within the cell, distinct from nuclear DNA. Damage to mitochondria can lead to the release of mtDNA into the cytosol. Furthermore, using MitoTracker Green FM staining, we observed that infection with EsxN-overexpressing bacteria resulted in an increase in mitochondrial mass or quantity (Fig. S5). This mitochondrial perturbation may ultimately affect mitochondrial function and the stability of mtDNA. Upon sensing this cytosolic dsDNA, cGAS catalyzes the synthesis of cGAMP. This second messenger then binds to and activates STING, triggering the downstream signaling cascade. The activated cGAS-STING signaling pathway induces the transcription of ISGs in the nucleus while antagonizing the transcription of pro-inflammatory cytokines. This cascade enhances mycobacterial intracellular survival, aligning with the known function of Esx-5 system substrates in facilitating mycobacterial immune evasion from phagosomes. Importantly, this process is regulated and facilitated by ISG15 (Fig. 8). However, it remains to be proven whether the change in the cytosolic dsDNA pool is directly and primarily contributed by lysosomal function. Future investigations should employ more precise methodologies—such as organelle-specific DNA labeling and tracking, membrane integrity assessment, and source-specific nuclease interventions—to further dissect the pathways of EsxN-mediated dsDNA release and its specific role in immune evasion.

Current research on EsxN primarily focuses on its potential as a subunit vaccine for tuberculosis treatment (43). By analyzing the mutation sites of EsxN from a database containing over 50,000 clinical *M. tuberculosis* mutant strains (12), we found that EsxN is highly conserved, with few clinical mutations. The high-frequency mutations of EsxN are almost synonymous mutations, indicating that EsxN is more inclined toward gene purification selection and also proving that EsxN is relatively stable and important in the genome (Fig. S6). This study delves deeper into the functional characteristics of EsxN in innate immunity, revealing that investigations into EsxN-host interactions are crucial for deciphering the mode of action of *M. tuberculosis* virulence factors and the infection outcomes in host cells. Notably, EsxN is secreted in complex with EsxM, another substrate of the Esx-5 system. EsxM has previously revealed important insights into pathogen evolution and host-pathogen interactions (44), which also tells that the functional interaction between these two is a key area of investigation, as their co-expression and potential synergistic effects may be crucial for virulence. We are currently exploring the immunomodulatory effects of the EsxM-EsxN complex, and preliminary data from their co-expression suggest synergistic effects, which will be the focus of future studies.

In viral infections, the MDA5 protein, localized in the cytoplasm and induced by IFN-β, contains a CARD domain and an RNA helicase motif (45, 46). It binds viral dsRNA, sensing viral replication intermediates (47). However, the induction of type I IFNs, important for host defense against viral infections, is ineffective in the context of bacterial pathogens (27), such as *M. tuberculosis*, and is associated with a greater extent of disease (48). Research indicated that a common IFN-β–inducible gene program correlates with the extent of disease in both leprosy and tuberculosis, suggesting that IFN-β is a common factor contributing to pathogenesis in the two distinct mycobacterial diseases (22). This is consistent with the expression trend of type I interferon-related genes observed in patients with active tuberculosis (Fig. 4A and B). Bacteria and viruses employ distinct replication strategies: bacteria can replicate asexually and independently, while viruses rely entirely on the host for replicating their genetic material. Consequently, the aforementioned protein (MDA5) cannot effectively target bacterial genetic material. This study focuses on the role of type I interferons in shaping the host infection process and outcome during bacterial infection, providing a more detailed exploration of the similarities and differences in type I interferon functions between viral and bacterial infections. The non-canonical dependency on ISG15 for EsxN-triggered dsDNA leakage presents a particularly intriguing layer of this manipulation. Traditionally, ISG15 is considered an antiviral ISG that can exert its effects through protein conjugation (ISGylation) or as a free cytokine. Our data suggest that EsxN exploits a previously unrecognized, STING-independent role of ISG15 in regulating organellar membrane stability or DNase activity. In this study, ISG15 is not a passive downstream effector molecule but a key amplifier that amplifies the initial dsDNA signal through its modifying activity, thereby establishing a positive feedback loop driving immunosuppression. This is a classic example of pathogens transforming host defense weapons into tools for their own survival. We propose a model where EsxN initiates a primary signal, which is then dramatically amplified by ISG15, potentially through the ISGylation of key proteins involved in lysosomal biogenesis, mitochondrial integrity, or DNA repair. This mechanistic insight places EsxN among a growing list of pathogen effectors that “hijack by activation,” turning the host’s most robust defense systems against itself.

Although some studies suggest that cGAS-STING pathway activation may be accompanied by downstream NF-κB activation, our results showed no significant difference in NF-κB activity attributable to EsxN overexpression (Fig. S5). We propose that EsxN strongly upregulates the type I interferon response, which in turn negatively regulates canonical pro-inflammatory pathways. This ultimately weakens the cell’s bactericidal inflammatory response, and the activation of NF-κB was delayed, which further enhances mycobacterial survival, exemplifying the counterbalancing mechanisms within immune signaling networks.

However, this study has certain limitations. We have not yet been able to precisely define the organelle origins of cytoplasmic dsDNA, and the function of EsxN in its native pathogen, *M. tuberculosis*, needs further validation in a BSL-3 environment. Nevertheless, this study provides a molecular and cellular framework for how a secreted *M. tuberculosis* protein, EsxN, participates in macrophage immune evasion, explaining how EsxN acts as one of *M. tuberculosis*’s virulence factors to modulate bacterial structure, immune cell responses, and its targets during infection. Therapeutically, targeting ISG15 or dsDNA leakage could disrupt this pathway, offering new host-directed therapies for tuberculosis. Additionally, EsxN’s conserved role supports its potential as a subunit vaccine candidate or diagnostic biomarker.

## Data Availability

The raw sequence data reported in this paper have been deposited in the Genome Sequence Archive (Genomics, Proteomics & Bioinformatics 2025) in National Genomics Data Center (Nucleic Acids Res 2025), China National Center for Bioinformation/Beijing Institute of Genomics, Chinese Academy of Sciences (GSA-Human: HRA015394) that are publicly accessible at https://ngdc.cncb.ac.cn/gsa-human/ (49, 50). All data generated or analyzed during this study are included in this published article and its supplemental material. Additional datasets are available from the corresponding author upon reasonable request.

## AFTER EPUB

*[This article was published on 25 February 2026 with errors in the Funding section. Funding was corrected in the current version, posted on 13 March 2026.]*

## SUPPLEMENTAL MATERIAL

Supplemental material spectrum.02488-25-s0001.pdf Fig. S1 to S6.

ASM does not own the copyrights to Supplemental Material that may be linked to, or accessed through, an article. The authors have granted ASM a non-exclusive, world-wide license to publish the Supplemental Material files. Please contact the corresponding author directly for reuse.
