# Supplementary material for: EsxN drives ISG15-mediated dsDNA release to activate cGAS-STING signaling and promote mycobacterial survival
Source: Microbiol Spectr. 2026 Feb 25;14(4):e02488-25. doi: 10.1128/spectrum.02488-25 (PMC13055286; doi:10.1128/spectrum.02488-25)

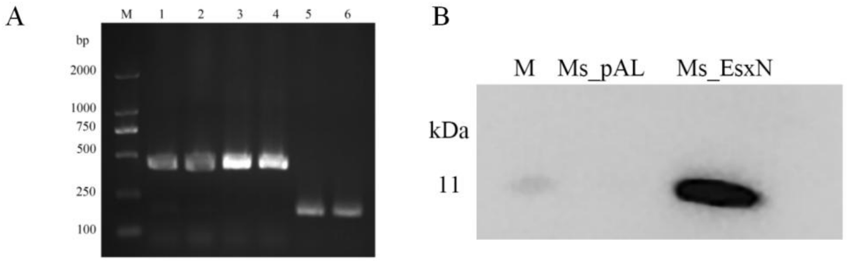

Supplementary Figure 1. A. Successful construction of Ms_EsxN and Ms_pAL, with lanes 1-4 of Ms_ EsxN, 5-6 is Ms_ pAL. B. The EsxN protein of Mycobacterium tuberculosis was heterologously expressed in Mycobacterium smegmatis, with an EsxN protein of about 9.9 kDa and His tag of about 0.84 kDa. Western blotting verified a fusion protein with a size of about 11 kDa.

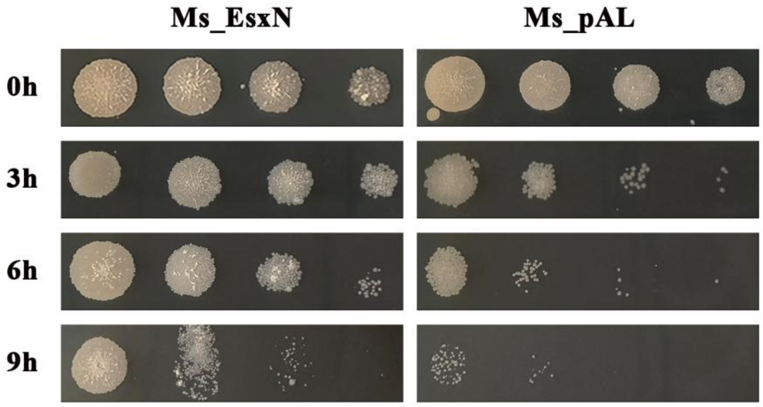

Supplementary Figure 2. Ms_ EsxN is more tolerant in acidic environments with pH=3 than Ms_ pAL. The Ms cultured in the environment with pH=3 are diluted in gradient, and the concentration is from 10-1 to 10- 4 for drip plates to observe the growth of Mycobacterium smegmatis.

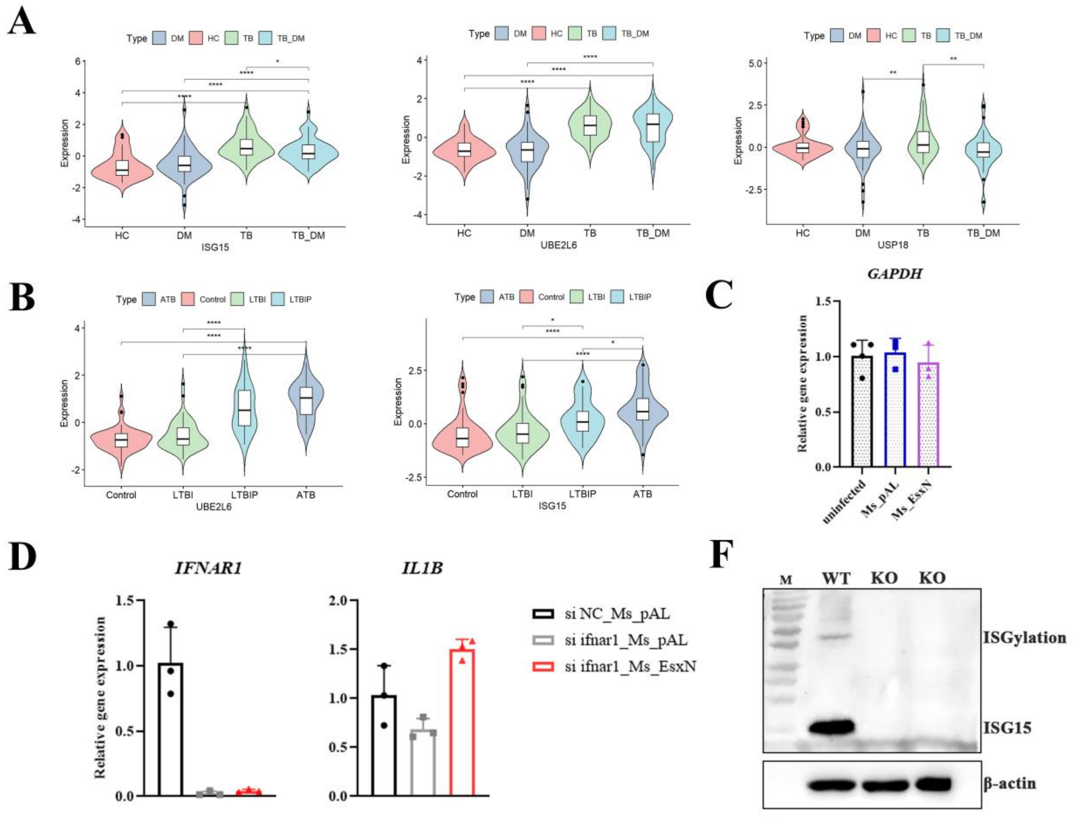

Supplementary Figure 3.

(A) The public clinical database GSE114192 of tuberculosis and diabetes patients from ncbi was analyzed. Violin plots of type 1 interferon-related genes were plotted using R Studio (version 4.4.1), and significance was analyzed based on LogFold Change.

(B) The public clinical database GSE107994 of tuberculosis patients from ncbi was analyzed. Violin plots of type 1 interferon-related genes were plotted using R Studio (version 4.4.1), and significance was analyzed based on LogFold Change.

(C) GAPDH as a second housekeeping gene for RT-qPCR is detected by R-qPCR.

(D) The pro-inflammatory immune level of the host and the transcription of IFNAR1 at the time points of 24 hours after silenced IFNAR1 and THP-1 infection were detected by RT-qPCR.

(E) Western blotting to verify ISG15 has been knockout successfully.

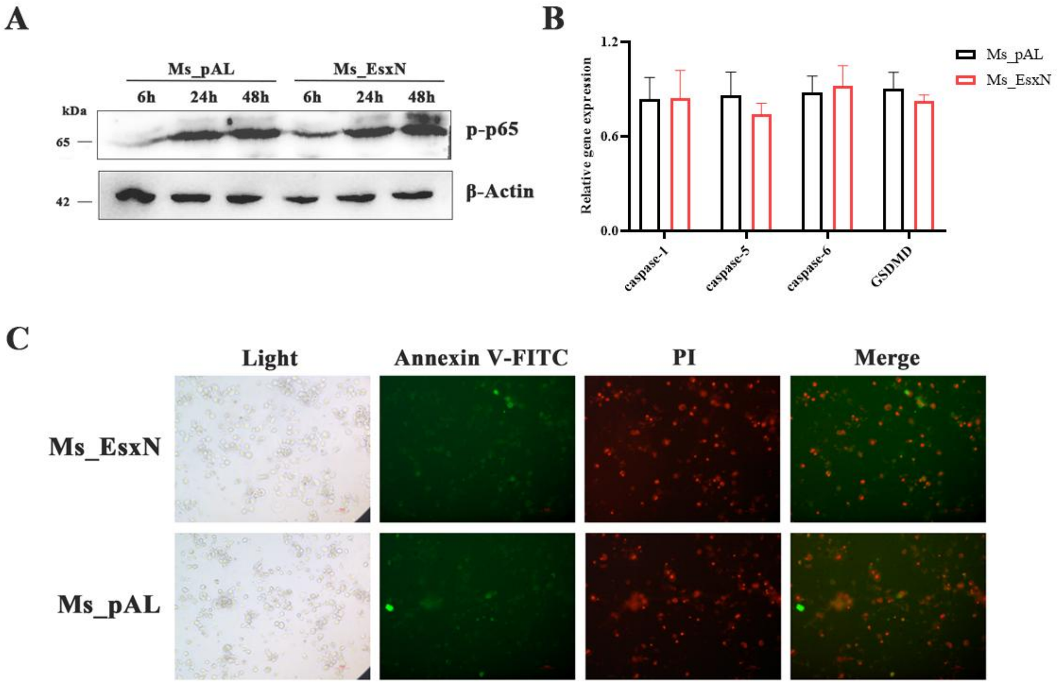

Supplementary Figure 4. (A) Western Blotting was used to detect the expressional levels of p-p65 in THP-1 cells infected with Ms_pAL and Ms_EsxN for 6h, 24h and 48h. (B) The transcriptional level of *caspase-1, caspase-5, caspase-6, GSDMD* in THP-1 cells infected 24 hours ago was detected by RT-qPCR. (C) Apoptosis levels in macrophages infected with the strains for 24 h were detected using a fluorescence microscope.

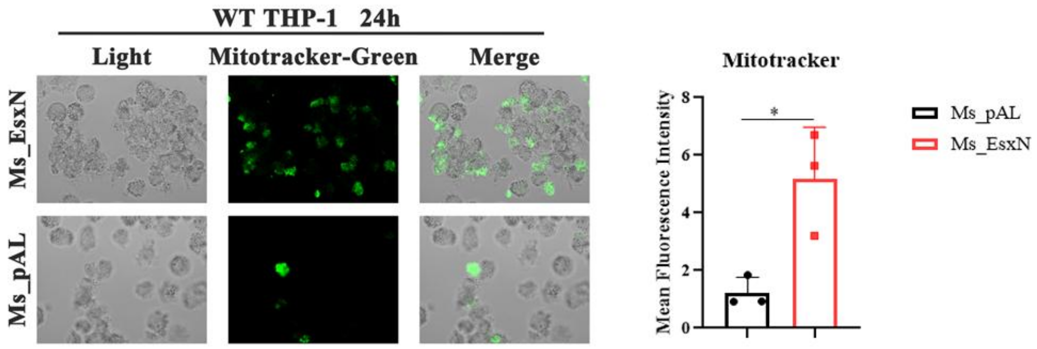

Supplementary Figure 5. Immunostaining of the content of intracellular mitotracker 24 hours after infection, which was captured by confocal fluorescence microscopy (100x). Statistical analysis of the mean fluorescence intensity (MFI) of MitoTracker staining in cells infected with MS in image J. All error bars represent standard deviation; (*P ≤ 0.05, **P ≤ 0.01,***P ≤ 0.001; n = 3).

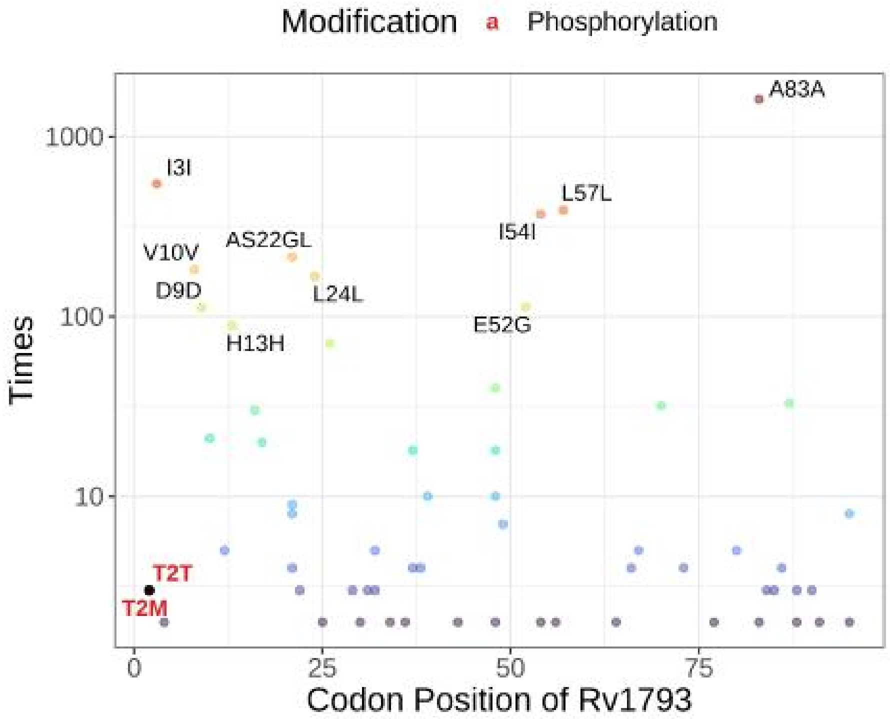

Supplementary Figure 6. The mutation sites of EsxN were analyzed from a database containing over 50,000 clinical *Mycobacterium tuberculosis* mutant strains, and the top ten high-frequency mutation sites were selected for scatter plots. Reference website: http://www.clipme.top:11725/.

Full uncropped Gels and Western Blot images

Figure 3C.

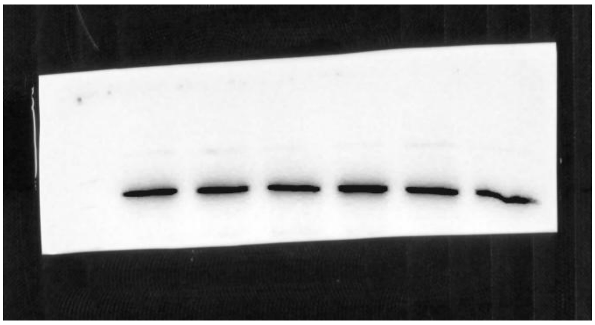

β-Actin (43kDa)

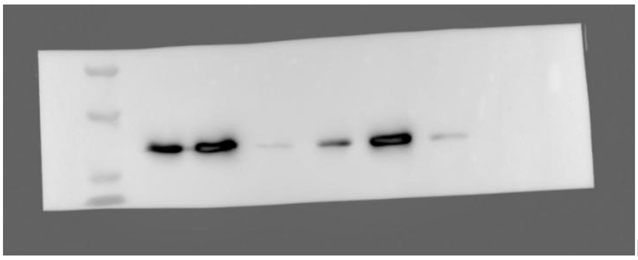

IL-1B (30kDa)

Figure 4G.

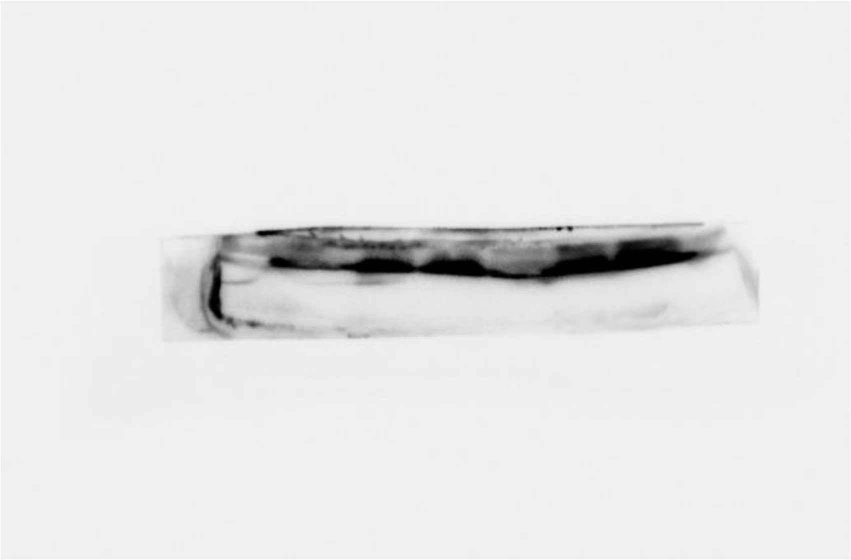

ISG15 (15kDa)

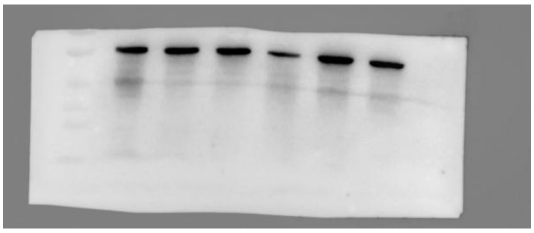

β-Actin (43kDa)

Figure 4G.

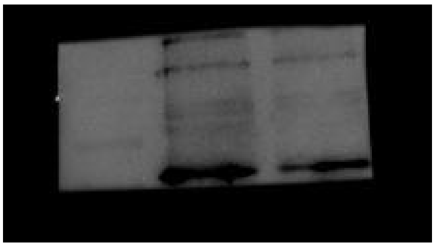

STAT1 (91kDa)

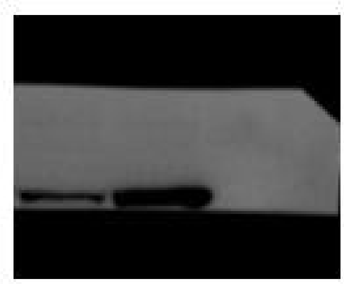

p-STAT1 (91kDa)

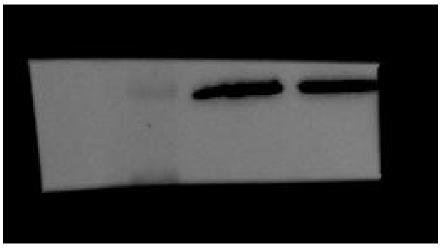

β-Actin (43kDa)

Figure 5A.

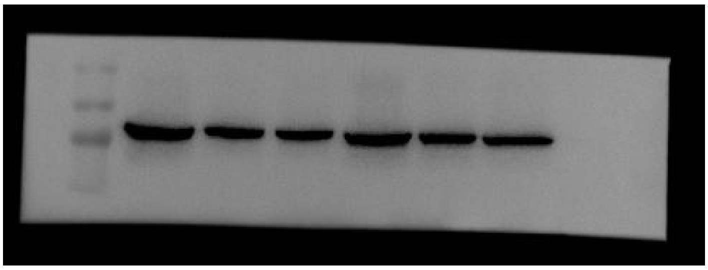

TBK1 (84kDa)

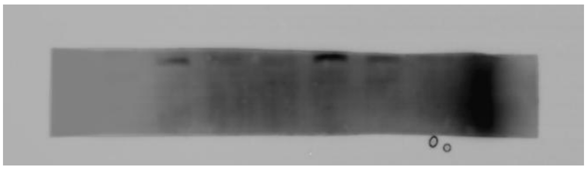

p-TBK1 (84kDa)

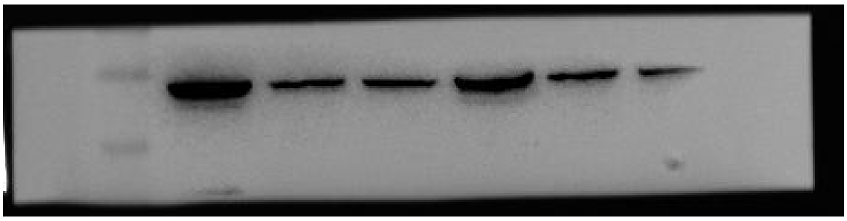

IRF3 (50kDa)

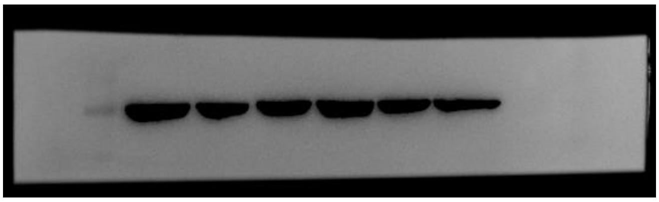

β-Actin (43kDa)

This β-Actin was used as a loading control for IRF3, TBK1 and p-TBK1, and they were from the same set of experiment.

Figure 6A.

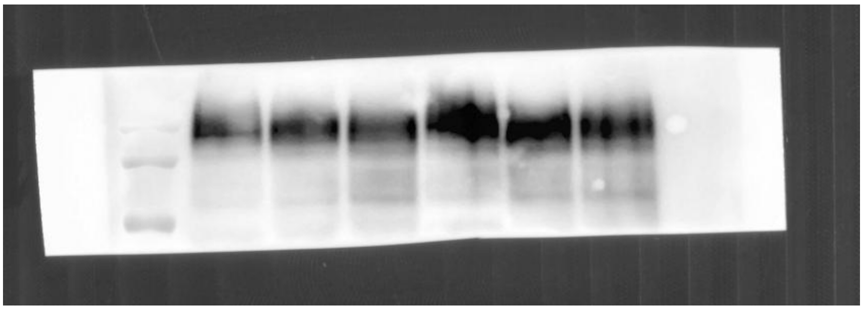

LAMP1 (90-120 kDa)

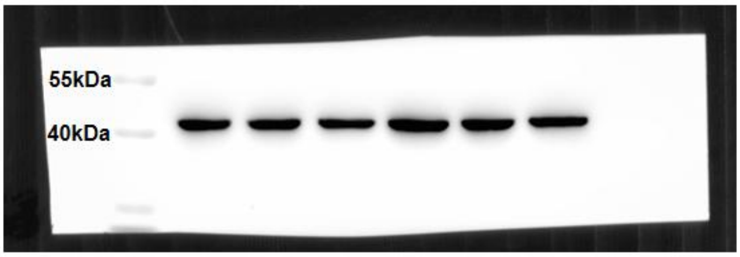

β-Actin (43kDa)

This β-Actin was used as a loading control for ISGylation and LAMP1 in supplementary figure 5, and they were from the same set of experiment.

Figure 7A.

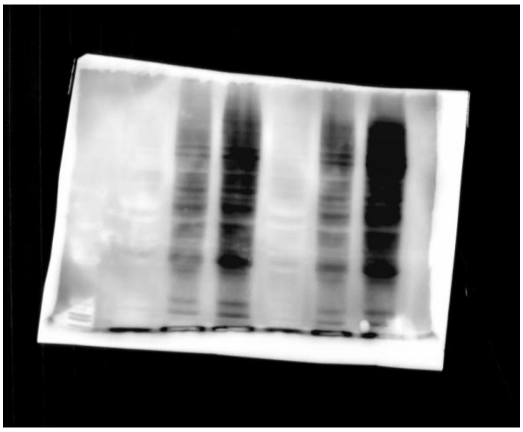

ISGylation (Full Gel)

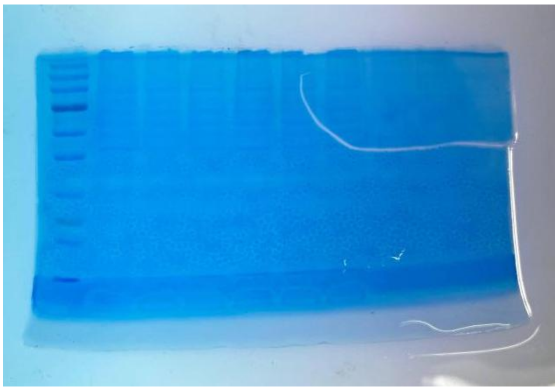
Commas Blue staining of total protein (Full Gel)

Supplementary Figure 1 B.

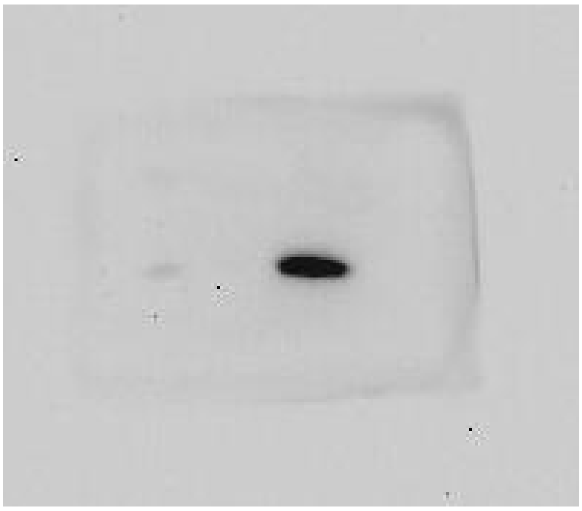
His-EsxN(10kDa)

Supplementary Figure 3.

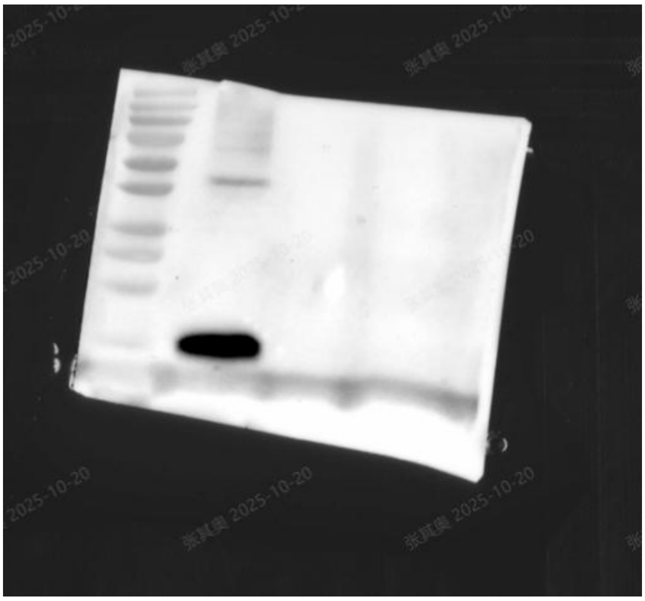
ISG15 (15kDa)

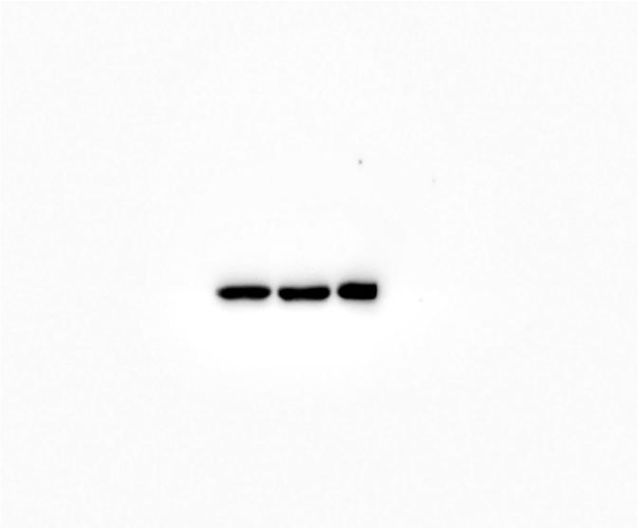

β-Actin (43kDa)

Supplementary Figure 4

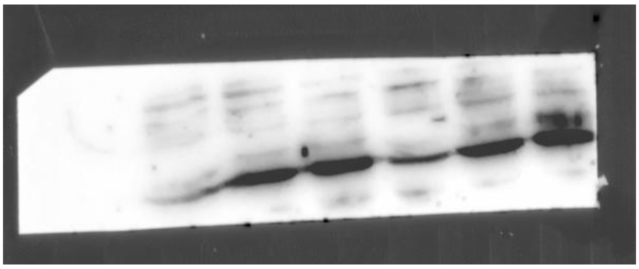

p-p65 (65kDa)

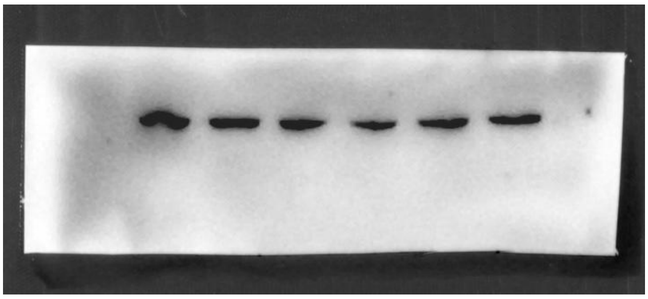

β-Actin (43kDa)

Full Gels and agarose gel electrophoresis images

Supplementary Figure 1 A.

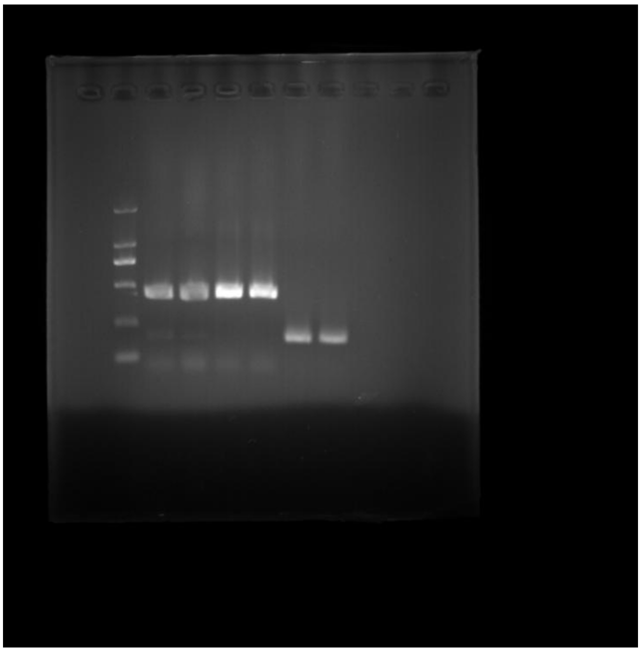

Supplement: Supplemental material — Fig. S1 to S6. [file spectrum.02488-25-s0001.pdf]
